# Benchmarking compound activity prediction for real-world drug discovery applications

**DOI:** 10.1038/s42004-024-01204-4

**Published:** 2024-06-04

**Authors:** Tingzhong Tian, Shuya Li, Ziting Zhang, Lin Chen, Ziheng Zou, Dan Zhao, Jianyang Zeng

**Affiliations:** 1 Institute for Interdisciplinary Information Sciences, Tsinghua University, Beijing, China; 2 Department of Automation, Tsinghua University, Beijing, China; 3 MOE Key Laboratory of Bioinformatics, Tsinghua University, Beijing, China; 4 Silexon AI Technology Co., Ltd., Nanjing, Jiangsu Province China; 5 Present Address: School of Engineering, Westlake University, Hangzhou, Zhejiang Province China

**Keywords:** Virtual screening, Cheminformatics, Screening, Computational chemistry

## Abstract

Identifying active compounds for target proteins is fundamental in early drug discovery. Recently, data-driven computational methods have demonstrated promising potential in predicting compound activities. However, there lacks a well-designed benchmark to comprehensively evaluate these methods from a practical perspective. To fill this gap, we propose a Compound Activity benchmark for Real-world Applications (CARA). Through carefully distinguishing assay types, designing train-test splitting schemes and selecting evaluation metrics, CARA can consider the biased distribution of current real-world compound activity data and avoid overestimation of model performances. We observed that although current models can make successful predictions for certain proportions of assays, their performances varied across different assays. In addition, evaluation of several few-shot training strategies demonstrated different performances related to task types. Overall, we provide a high-quality dataset for developing and evaluating compound activity prediction models, and the analyses in this work may inspire better applications of data-driven models in drug discovery.

## Abstract (web summary)

Data-driven computational methods have demonstrated promising potential in predicting compound activities from chemical structures, however, unbiased practical applications remain challenging due to the lack of proper benchmarking methods. Here, the authors develop a benchmark termed CARA to eliminate the biases in current compound activity data by using new train-test splitting schemes and evaluation metrics, revealing accurate and informative model performances.

## Introduction

Predicting the biochemical activities or binding affinities of compounds against protein targets is of vital importance in modern target-based drug discovery^1,2^. Most of the currently identified drug targets are proteins, and small-molecule compounds still account for more than 70% of approved drugs^3^. For a typical drug discovery pipeline, there are several stages associated with compound activity characterization and prediction, including hit identification, hit-to-lead optimization, and lead optimization stages^4^. In the hit identification stage, in general, a small number of active compounds (i.e., hit compounds) are discovered from large-scale chemical libraries as the starting points for further drug design and optimization^5^. Characterizing the activities of candidate compounds is the major issue in this stage, and virtual screening (VS) is often employed to increase the success rates and efficiency and reduce the cost of experimental screening^6^. In the hit-to-lead or lead optimization stage, the activities of candidate compounds need to be further optimized to ensure that they can possibly reach the desired efficacy in the subsequent pre-clinical and clinical experiments^4^. Although other aspects including pharmacokinetic parameters and safety issues are also considered in this stage, the binding activities are always one of the indispensable properties^7^. In this stage, medicinal chemists may focus on the rankings of activities among a series of specially designed compounds, and investigate the structure-activity relationships for designing better molecules. Computational methods for addressing the compound activity prediction problem have also been applied to assist this drug discovery stage^8,9^.

The compound activity prediction methods can be generally classified into two main categories, i.e., knowledge-based and data-driven methods. Traditional knowledge-based computer-aided drug design (CADD) mostly depends on chemical and physical rules and empirical assumptions^6^. The commonly used CADD methods, including molecular docking and molecular dynamics simulation, can be applied to estimate the binding energies and dynamics between compounds and proteins^10^. Despite their advantages in relatively good interpretability, the limited precision and high demand on the computational resources are the major concerns of the applications of these methods^11^. On the other hand, recent emerging data-driven methods, including machine learning and deep learning-based methods, also sometimes referred to as artificial intelligence (AI)-based methods, have exhibited better accuracy with a relatively lower requirement of computational resources in a number of compound activity prediction scenarios^12^.

The prediction ability of data-driven methods depends on accurately learning the underlying patterns from large-scale and high-quality data. Since the binding affinities or activities between compounds and proteins need to be measured through biophysical, biochemical, or cell-based experiments^13^, it is generally difficult to obtain large-scale and high-quality compound activity datasets in individual studies. Fortunately, public resources, including ChEMBL^14^, BindingDB^15^, and PubChem^16^, have provided access to the massive amounts of compound activities from a large number of previous studies.

There exist a number of benchmark datasets that are frequently adopted by data-driven methods, including Davis^17^, KIBA^18^, DUD-E^19^, MUV^20^, BindingDB^21^, *human*^22^, *C.elegans*^22^, PDBbind^23^, and recently proposed FS-Mol^24^. However, there are still gaps between these datasets and the desired ones for training and evaluating the data-driven models. Through carefully analyzing the characteristics of real-world compound activity data from previous studies, we found that the data distributions of existing benchmark datasets do not completely match the real-world scenarios, in which the experimentally measured data are generally sparse, unbalanced, and from multiple sources. DUD-E^19^ introduces simulated compounds (i.e., decoys) for molecular docking to enhance the benchmark dataset. However, the generated decoys can be of lower confidence for evaluation and may introduce bias because the actual activities are not measured^25^. MUV^20^ proposes a maximum unbiased validation dataset for virtual screening by considering the characteristics such as similarities between compounds and diversity of chemical space. Nevertheless, the decoys are still introduced as inactive compounds and may also cause bias. Davis^17^ focuses on the activity and selectivity of 72 kinase inhibitors against 442 kinases, in which unfortunately the targets are only a special class of proteins and the number of compounds is relatively small. In some structure-based datasets, such as PDBbind^23^, though containing compound activities against certain protein targets, the number of ligand compounds per target is limited and does not reflect the practical cases. FS-Mol^24^ derives a dataset of molecules with experimentally measured activities for quantitative structure-activity relationship (QSAR) analyses. However, it simply excludes the assays from the high-throughput screening (HTS) only based on the numbers of data points. Also, only a simple binary classification task is employed in this benchmark, which could be less generalizable to real-world applications as the rankings of positive samples are generally more important in practice. In addition to the characteristics of data, the evaluation schemes applied to these datasets are not able to completely reveal the prediction abilities of computational models in various application scenarios of compound activity prediction. As a result, constructing a benchmark dataset using the data from real-world applications to meet the practical requirements is fundamentally important for developing and evaluating different compound activity prediction models.

To address this problem, we curated a benchmark of compound activity prediction for real-world applications, named CARA, based on the characteristics of real-world data. We first distinguished the compound activity data into two application categories, i.e., virtual screening (VS) and lead optimization (LO), corresponding to the two types of drug discovery tasks. We then designed the data splitting schemes especially for the two task types and unbiased evaluation approaches to provide a comprehensive understanding of model behaviors in practical situations. We also considered two situations when a few samples are already measured, termed few-shot scenario, and no task-related data are available, termed zero-shot scenario, to account for different application settings. Typical types of state-of-the-art machine learning and deep learning methods, as well as several classical training strategies, were comprehensively evaluated on our CARA benchmark.

Through comprehensive analyses, we discovered that popular training strategies such as meta-learning^26^ and multi-task learning^27^ were effective for improving the performances of classical machine learning methods for the VS tasks. In contrast, training the quantitative structure-activity relationship models^28^ on separate assays already achieved decent performances in the LO tasks. Further analyses demonstrated that different training strategies in the few-shot scenario were preferred for the VS or LO tasks possibly due to the distinct data distribution patterns of the two tasks. In addition, we identified the accordance of outputs between different models as a useful indicator to estimate the model performances even without knowing the activity labels of test data. We also revealed the limitations of current computational models in sample-level uncertainty estimation and activity cliff prediction. In summary, CARA can serve as a practically useful benchmark for developing, evaluating, and understanding current computational models for compound activity prediction, and thus provide helpful guidance for applying these models to real drug discovery applications.

## Results

### Characteristics of compound activity data in real-world applications

**Fig. 1:**
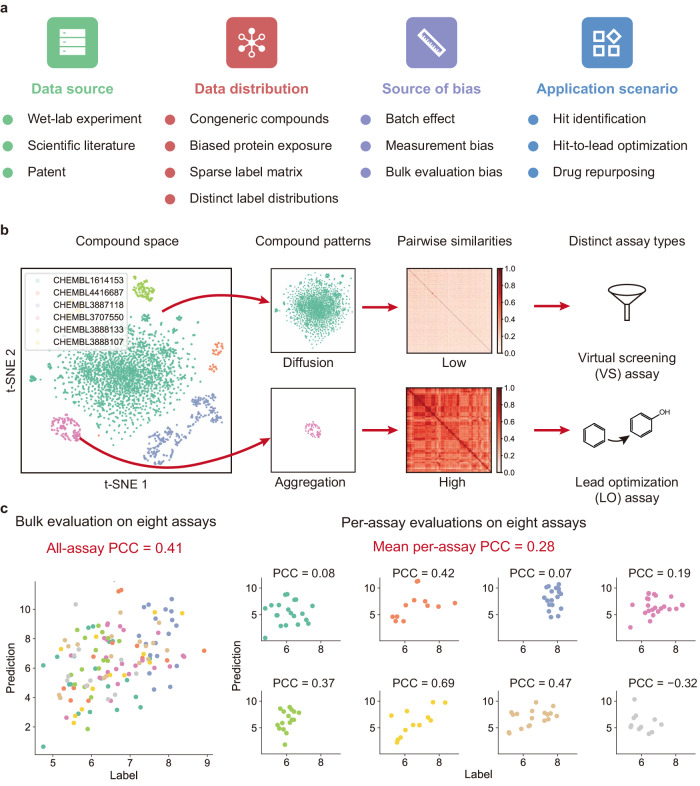
Characteristics of current compound activity data in real-world applications. **a** The summarized characteristics or problems of real-world compound activity data in four aspects. **b** Visualization of compound distributions on several example assays in the ChEMBL database through t-distributed stochastic neighbor embedding (t-SNE) on molecular fingerprints. Based on the pairwise similarity patterns of compounds, the assays can be distinguished into two categories, i.e., the virtual screening (VS) assays with dissimilar compounds, and the lead optimization (LO) assays with congeneric compounds. **c** Illustration of the bulk evaluation bias on the simulated data. The performances were evaluated in terms of Pearson’s correlation coefficients (PCCs). The bulk evaluation (all-assay) using the data of all assays can be overestimated compared with those on individual assays (per-assay), and thus cannot reflect the real performance. The meaningless predictions with nearly zero or even negative PCCs can be hidden by the bulk evaluation strategy. For the eight simulated assays, the predictions and true labels are plotted together (bulk) or separately (per-assay).

To develop a benchmark dataset according to the practical situations for compound activity prediction, we first analyzed the characteristics of available activity data generated in current real-world drug discovery process. The ChEMBL database^14,29^ provides millions of well-organized compound activity records from previous scientific literature, patents, and other public resources. These activity data can be grouped according to the ChEMBL Assay ID labels, so that each group contains a set of compound activities against a target protein measured under a specific experimental condition from the same data source. To be brief, we use the term assays to describe such groups of activity data (see an example of an assay shown in Supplementary Table 1). An assay is defined as a collection of samples with the same protein target and measurement conditions but associated with different compounds. A sample contains the activity value of a compound against a protein target measured in certain conditions. In particular, each assay represents a specific case of the drug discovery process in which the protein-binding activities of a set of compounds were measured. As a result, analyzing the characteristics of these assays can provide a comprehensive and practical understanding of the compound activity data. According to our careful analyses, we observed that there were several typical characteristics or problems of the current real-world compound activity data (Fig. 1a and Supplementary Table 2).

#### Multiple data sources

The compound activity data from the ChEMBL database^14^ were all from wet-lab experiments organized according to assays. These assays can well reflect a broad range of real-world application scenarios in the drug discovery process and provide sufficient information for the investigation. However, these data are from multiple sources, such as scientific literature or patents, and generated by different experimental protocols. Therefore, further efforts are needed to carefully examine their data distributions and potential biases before integrating them for objectively evaluating different prediction models.

#### Existence of congeneric compounds

Through visualizing compounds in different assays using t-distributed stochastic neighbor embedding (t-SNE^30^, Fig. 1b), we found that compounds from different assays exhibited two main distinct patterns: one is diffused and widespread, while the other is aggregated and concentrated. Further analyses revealed that the compounds in the assays with diffused patterns had relatively lower pairwise similarities, while the compounds in the assays with aggregated patterns were much more similar to each other (examples shown in Fig. 1b). Such differences in the two types of assays may be explained by the different drug screening and designing strategies in the two stages of drug discovery. More specifically, in the hit identification stage, a hit compound is generally screened from a large and diverse compound library^5^, resulting in a diffused distribution pattern. On the other hand, in the hit-to-lead or lead optimization stage, a series of congeneric compounds are often designed based on several already discovered starting points (i.e., the hit or lead compounds)^31^. Therefore, compounds in the series often share similar scaffolds or substructures and thus exhibit relatively high similarities to each other.

According to the pairwise similarities of compounds in the same assay, we classified the assays into two types, named virtual screening (VS) assays and lead optimization (LO) assays, which were corresponding to the assays with diffused and aggregated compound distribution patterns, respectively. The existence of congeneric compounds makes the LO assays become a quite distinct type of activity prediction tasks compared to the VS assays, which means that the VS and LO assays should be considered separately when constructing a practical compound activity benchmark for evaluating the corresponding prediction tasks.

#### Biased protein exposure

There were thousands of proteins targeted by small molecules in the ChEMBL database^14^. However, these proteins were not evenly explored in the previous studies. Here, we used the number of assays related to a protein target to approximately reflect its degree of exposure. In the ChEMBL database, about 39% and 35% of the proteins were investigated only once in the VS and LO assays, respectively, while the most popular target was tested over 900 times (Supplementary Fig. 1a, b). When evaluating the generalization ability of a compound activity prediction model, the performance metrics were expected to reflect the overall prediction power across all types of protein targets. Therefore, without the proper design of a benchmark dataset for performance evaluation, the long-tailed distribution of protein exposure (i.e., the occurrence of a protein in the dataset) may introduce biases towards those frequently investigated protein targets.

#### Sparse label matrix

By filling the activity labels into a matrix with compounds in the columns and proteins in the rows, we observed that the label matrix exhibited a sparse pattern, in which most of the elements remained unknown (Supplementary Fig. 1c). In fact, a protein target is often tested with a number of compounds in the drug discovery process but compounds are seldom explored with multiple targets. On average a compound was tested about four times (317,855 unique compounds in 1,237,256 samples) in the VS assays and twice (625,099 unique compounds in 1,187,136 samples) in the LO assays (Supplementary Table 3). In particular, a dense label matrix is generally difficult to obtain. Therefore, training or evaluating models on datasets adopting a dense label matrix may not generalize well to real-world applications.

#### Distinct label distributions

Previous studies stated that there were more positive samples (active compounds) than negative samples (inactive samples) in compound activity datasets since the inactive compounds were seldom reported^32,33^. This was true if all the assays were considered together. However, we made different conclusions when looking into the VS and LO assays separately. Here, we adopted the activity label in the logarithm scale, which is also referred to as p(activity) value and defined as $-{\log }_{10}$(activity [mol/L]), where the activity can be measured by half maximal inhibitory concentration (IC_50_), half maximal effective concentration (EC_50_), inhibition constant (K_i_), dissociation constant (K_d_), or potency. Our analyses revealed several characteristics about the distributions of activity labels in both VS and LO assays. First, the distributions of activity labels were different between VS and LO assays. The shape of label distribution for the VS assays was skewed (Supplementary Fig. 1d) while that for the LO assays was nearly symmetric (Supplementary Fig. 1e). For most individual VS and LO assays, their labels were also distributed in such patterns (examples shown in Supplementary Fig. 1f, g). Second, if we took the commonly used threshold with the p(activity) value of 6 (corresponding to the activity label of 1 μmol/L) to distinguish positive and negative samples^19^, there were fewer positive samples than negative ones in the VS assays, but more active samples than inactive ones in the LO assays (Supplementary Fig. 1d, e). In addition, the positive-to-negative ratios may vary in different individual assays (Supplementary Fig. 1f, g), suggesting that a universal threshold may not suit well for all the assays. Since both assay types (i.e., VS or LO) and characteristics of individual assays can influence the label distributions, these factors should be considered when designing the evaluation metrics of activity prediction models.

#### Measurement bias and batch effect in assays

Different assays may adopt different experimental techniques and conditions, thus resulting in assay-specific batch effects for the corresponding labels. We first examined the distributions of assay labels with different measurement types, such as IC_50_ and K_i_. As illustrated in Supplementary Fig. 1h, i, for both VS and LO assays, the activity values measured by pIC_50_ (i.e., the $-{\log }_{10}$(IC_50_ [mol/L])) tended to be smaller than those by pK_i_ (i.e., the $-{\log }_{10}$(K_i_ [mol/L])) on the same compound-protein pairs, which can be partly explained by their known relationship in typical enzyme-inhibitor interactions^34^, that is, ${{{{{{{{\rm{pIC}}}}}}}}}_{50}={{{{{{{{\rm{pK}}}}}}}}}_{{{{{{{{\rm{i}}}}}}}}}-{\log }_{10}({{{{{{{\rm{S}}}}}}}}/{{{{{{{{\rm{K}}}}}}}}}_{m}+1)$, where S denotes the substrate concentration and K_*m*_ denotes the Michaelis-Menten constant. Similar measurement bias can also be observed in other measurement types as shown in Supplementary Fig. 1j. Despite the measurement types, the experimental conditions can also affect the distributions of the compound activity labels. For example, there was obvious bias among the affinities measured in different batches of the same set of compound-protein pairs even with the same measurement type^35^ (Supplementary Fig. 1k). These observations indicated that it is generally not suitable to directly combine the activity labels of samples from different assays together for the downstream model evaluation process.

#### Bulk evaluation bias

Performance evaluation of a compound activity prediction model is usually performed on all the test data. If the test dataset contains activities from different assays with distinct ranges, it is possible to cause biased evaluation. For example, as shown in a simulated task (Fig. 1c) containing eight assays, the bulk evaluation considering all the assays achieved a Pearson’s correlation coefficient (PCC) of 0.41. However, when considering the performances of the eight assays individually, the average per-assay PCC was only 0.28, which was significantly lower than the all-assay PCC. In addition, the per-assay PCCs ranged from − 0.32 to 0.69 in this example, with four out of the eight assays having poor PCCs (i.e., lower than 0.2). Such a phenomenon can be partly explained by the fact that the assay actually acts as a confounding factor between predictions and labels. In other words, the labels of the eight assays were not identically distributed. As shown in Fig. 1c, the labels of some assays were relatively higher than those of the other assays, exhibiting diverse distributions. Therefore, by combining the sample points of those assays that were poorly predicted but with different label distributions, the overall correlation can be overestimated if the model was good at distinguishing assays (actually proteins) instead of compounds.

#### Diverse application scenarios

There are several stages in the drug discovery and development process that are associated with compound activities, such as hit identification, hit-to-lead optimization, lead optimization, and drug repurposing. These stages generally focus on different perspectives of compound activities. For example, in the hit identification stage, a hit compound with fair activity at the micromolar concentration is generally sufficient. However, in the hit-to-lead optimization or lead optimization stage, a compound with better activity at the nanomolar concentration is usually required. In addition, the distributions of candidate compounds are generally different for these stages. In the hit identification stage, a library containing a large variety of compounds is usually preferred while in the optimization stages, congeneric compounds are often designed and tested. For drug repurposing, in general, the candidate compounds are from approved or investigated drugs with good properties. Therefore, designing special benchmark datasets for different application scenarios of compound activity prediction is needed.

In summary, all the above characteristics of real-world compound activity data need to be taken into consideration when designing a benchmark dataset for evaluating different data-driven compound activity prediction models. By doing so, we believe that the bias in the data can be avoided and the gap between current real-world data and practical applications can be bridged, which can thus help better estimate the model performance and guide the model development.

### CARA: a practical compound activity prediction benchmark for real-world applications

Here, we propose a benchmark dataset, named CARA, for developing and evaluating computational methods for Compound Activity prediction in Real-world Applications by considering the characteristics of real-world activity data and designing proper data splitting schemes and evaluation metrics. We introduce the design of CARA from the following three aspects:

#### Data curation

**Fig. 2:**
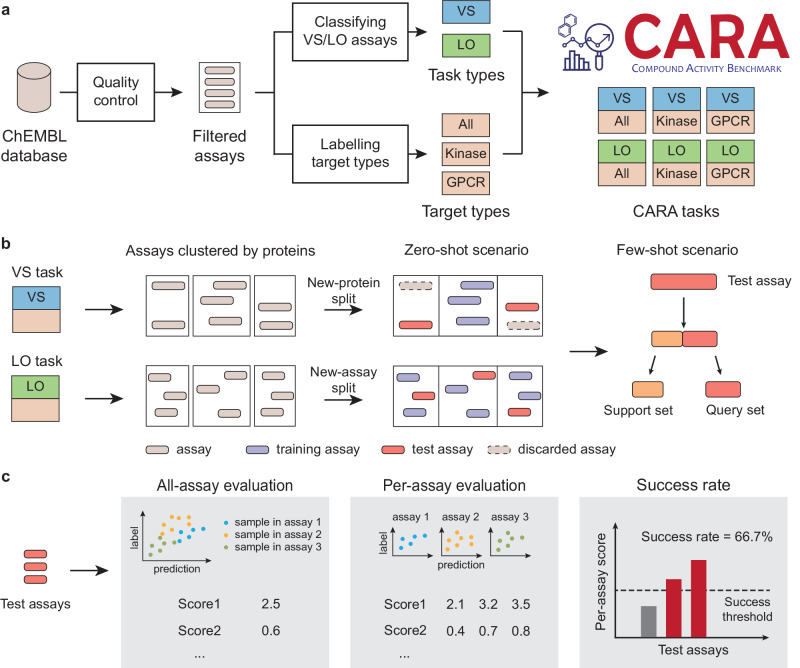
Overview of the CARA benchmark. **a** The pipeline of data curation from the ChEMBL database. The assays in the ChEMBL database were first filtered by a quality check process. Then, the assays were classified into two task types (i.e., VS or LO) according to their compound distributions. Assays were also labeled with the target types, including all proteins, kinases, and G protein-coupled receptors (GPCRs), denoted as All, Kinase, and GPCR, respectively. The CARA benchmark finally consisted of six tasks with two task types and three target types. **b** Data splitting schemes for VS and LO tasks. The assays were split into training and test assays through different schemes for the VS or LO tasks. More specifically, a new-protein split scheme was adopted for the VS tasks because hit identification is generally applied for novel proteins. For the LO tasks, on the other hand, a new-assay splitting scheme was used because the lead optimization process generally focuses more on a new scaffold or a new series of congeneric compounds. In addition, two scenarios named zero-shot and few-shot were further considered in CARA which corresponded to the scenarios where no and some relevant data were available, respectively. For the zero-shot scenario, the compound activity prediction model is supposed to be trained on a set of training assays and evaluated on an independent set of test assays. For the few-shot scenario, in addition to the training assays, a portion of the samples in the test assays is marked as labeled data (i.e., support set) and then used for model training or fine-tuning. The remaining samples of test assays (i.e., denoted as the query set) were used for performance evaluation. Here, 50 samples in each test assay were randomly selected as the support set. **c** Evaluation metrics of the three levels, i.e., all-assay evaluation, per-assay evaluation, and success rates. For all-assay evaluation, the evaluation metrics are calculated through comparing the predictions and true labels of all the test assays. This is not recommended for performance evaluation due to the bulk evaluation bias mentioned in the main text. For per-assay evaluation, the evaluation metrics are computed for each assay individually, resulting in distributions of performances over assays. The per-assay evaluation can provide a distribution of assay-level performances instead of a scalar value as in all-assay evaluation. The success rate can be obtained through calculating the percentage of successful assays defined by the per-assay metrics over a specific threshold. The success rate can provide a straightforward understanding of the performance of a model from the perspective of real-world applications.

CARA was curated mainly based on the ChEMBL^14^ database, which contained large-scale activity data of small molecule compounds against the corresponding protein targets. The pipeline of generating the CARA dataset mainly contained three steps (Fig. 2a). First, the assays in the ChEMBL database were filtered according to several criteria to maintain high-quality data (see “Methods” section). The ChEMBL assays containing multiple measurement types (e.g., IC_50_ and K_i_) were split so that a single assay defined in CARA contained compound activity data with only one measurement type. Second, the assays were classified into VS and LO assays based on the pairwise similarity patterns of compounds within the same assay. We used an empirical threshold of median pairwise similarities of 0.2 to distinguish the VS and LO assays according to our preliminary analysis (see “Methods” section). Third, since popular target types such as kinases or G-protein coupled receptors (GPCRs) are often specially considered in drug discovery scenarios, we particularly extracted the related data to form the corresponding prediction tasks for these two protein families^5^. We also included a target type containing all protein families (i.e., denoted as All) for general evaluation. In the end, our CARA benchmark contains two task types (i.e., VS or LO) and three target types (i.e., All, Kinase, or GPCR), resulting in six different activity prediction tasks (i.e., VS-All, LO-All, VS-Kinase, LO-Kinase, VS-GPCR, and LO-GPCR). More details about constructing our CARA benchmark can be found in Methods. Through comparing with typical drug discovery datasets, we found that the compounds in CARA can capture a considerable fraction of the drug-related chemical space (Supplementary Note 1 and Supplementary Fig. 12).

#### Train-test data splitting schemes

For both VS and LO tasks, the data were split for training and evaluation at the level of assays, which were termed training and test assays, respectively (Fig. 2b). In order to eliminate the protein exposure bias, the test assays were selected to contain diverse proteins. More specifically, all the assays were first clustered based on the protein similarities, and at most one assay in each cluster was selected into the test data. Then the training assays were selected from the remaining assays. In particular, for VS tasks, the assays in the protein clusters different from those of the test assays were selected as the training assays. In other words, the training and test assays shared different protein clusters. Such a data splitting strategy was termed the new-protein splitting scheme, which was suitable for VS tasks as screening a library of diverse compounds is generally conducted for those targets without any known active compounds. For LO tasks, all the remaining assays were selected as the training assays, which was termed the new-assay splitting scheme. Such a splitting strategy is suitable for LO tasks as the lead optimization tasks generally require a number of known starting points for the investigated targets. The new-assay splitting scheme for LO tasks was closely related to the previously used new-compound splitting strategy^36,37^, which split the training and test data according to compound identities or similarities. To prevent data leakage, the samples (i.e., compound-protein pairs) that overlapped with those in the test assays were removed from the training assays. Such data splitting schemes can guarantee the reliable extrapolation of the findings and insights obtained from our CARA benchmark to novel targets or compounds. In other words, the model performances evaluated under these splitting strategies can be used to predict their behaviors on newly discovered targets and compounds in the future, even if they may not have been covered by existing databases.

We also considered two practical scenarios for applying compound activity prediction methods on the CARA benchmark. In practice, compound screening or lead optimization processes are often conducted in multiple iterations. As a result, the compound activity prediction task could be benefited from the labeled data of the same assay from previous iterations. Here, we termed a setting the few-shot learning scenario^38^ when the labels of a small number of compounds in the test assays had already been measured. In comparison, a zero-shot learning scenario can be defined as the setting when no activity label was observed for the test assays, and the computational models were expected to make predictions for the novel assays. The goal of the few-shot scenario was to take advantage of a small subset of labeled samples to make better predictions for the remaining test samples. Therefore, in the few-shot scenario, samples in the test assays were further split into a support set and a query set. The support set with observed activity labels was applied for further optimizing the assay-specific models, and the query samples were used for performance evaluation (Fig. 2b).

#### Evaluation metrics

While most of the previous works ignored the difference between VS and LO assays and applied the same evaluation metrics to the compound activity prediction models, we noticed that the focus of such evaluation may change in different stages of the real drug discovery process. In the hit-identification stage (corresponding to the VS assays), the computational methods are generally applied to narrow down the range of compound candidates that need to be experimentally tested, thus saving time and cost^39^. Therefore, the accuracy of the top-ranked compounds is usually more important than the overall ranking accuracy of the whole compound library. Accordingly, we mainly calculated the enrichment factor (EF) for VS assays, which measured the accuracy of top-ranked compounds compared to random predictions. In contrast, during the hit-to-lead or lead optimization stages (corresponding to the LO assays), accurately ranking the activities of a series of congeneric compounds is often required. We thus used PCC as the major evaluation metric for LO assays.

Three levels of evaluation were performed and illustrated in the CARA benchmark (Fig. 2c). First, bulk evaluation on all the samples in the test assays was conducted in the zero-shot scenario. That is, the performance metrics were calculated by taking all the test assays as a whole set. However, the bulk evaluation was not adequate for performance evaluation and comparison due to its possibility of overestimating the practical model performances as described in the previous section. Therefore, we recommend the more informative per-assay metrics as the main criteria for performance evaluation. The per-assay metrics were calculated on individual test assays (i.e., one value for each test assay), and the distribution of these metrics over all the test assays was considered in performance comparison. In addition, the success rates (SRs) of activity prediction models were defined as the proportion of successfully predicted assays among all the test assays. More specifically, at least one hit compound ranked in the top 1% predictions and PCC no less than 0.5 were considered a successfully predicted assay for VS and LO tasks, respectively.

To summarize, we made the following major efforts to develop CARA as a practical compound activity benchmark (the characteristics of CARA compared with other compound activity datasets are summarized in Supplementary Table 4).

First, the compound activity data from wet-lab experiments were organized at the level of assays. These assays not only provided the training datasets but also can be regarded as individual case studies in real-world applications and thus can provide a direct evaluation of the learning tasks.

Second, we considered the VS and LO tasks separately to account for the difference in these two types of application scenarios of the drug discovery process. By distinguishing the compound activity data according to their task types, the distinct distributions of compounds or labels could be handled separately. Different train-test splitting schemes and metrics were also specially designed according to their purposes for evaluating VS and LO tasks.

Third, evaluation using per-assay metrics enabled one to estimate the performances with the distribution of scores over assays instead of a single score. Meanwhile, the success rates based on the per-assay metrics can provide the overall evaluation of a learning task. Assay-level evaluation can also help address the problems arising from the protein exposure bias, sparse label matrix, measurement bias, batch effect, and bulk evaluation bias which often happen when considering multiple assays together.

Fourth, in addition to the zero-shot scenario, the few-shot scenario was also supported by CARA for the situations when characterizing compound activities with assay-specific data from previous iterations. As a result, the CARA benchmark can be used to evaluate not only individual compound activity prediction models but also different few-shot training strategies.

As a result, the issues summarized in the previous section can be addressed by the above designs of CARA. First, we organized the compound activities in the ChEMBL database into individual assays, in which all the data points in a single assay were ensured to share the same protein target, experimental condition, measurement type, and data source. Second, we proposed to assess the performance of compound activity prediction methods by a per-assay evaluation scheme. Combining the above two designs, the previous issues including multiple data sources, measurement bias batch effect in assays, and bulk evaluation bias can be addressed. Third, we categorized the assays according to their data distribution into VS and LO to mimic different application scenarios in the early drug discovery process. The label distributions from VS and LO assays were also different according to our observation (Supplementary Fig. 1d, e). Therefore, by considering the VS and LO assays separately, the issues of existence of congeneric compounds, distinct label distributions, and diverse application scenarios can be properly taken into account. To consider biased protein exposure, we have classified the proteins into different protein classes (i.e., Kinase, GPCR or All) to formulate different datasets (Fig. 2a). More importantly, we clustered the assays in each dataset based on the similarities of protein sequences (Fig. 2b). When constructing the test assays, we selected at most one assay for each protein cluster such that the proteins in the test sets were not biased towards those frequently investigated targets. In addition, our assay-based organization of experimentally measured activity data can maintain the patterns of the sparse labels from real-world activity data, which enables the direct evaluation of the model performances on the currently available large-scale data.

### Performance evaluation of compound activity prediction models under the zero-shot scenario

First, we aim to conduct a comprehensive evaluation of deep learning-based models in predicting compound activities on CARA under the zero-shot scenario, i.e., without using labeled data for the test assays. For the VS assays which are split into training and test datasets according to protein similarities, the corresponding zero-shot scenario evaluates the model performances on novel proteins without any observed compound activity data. For the LO assays, the corresponding zero-shot scenario mimics the situation of evaluating newly-designed compound series, and the protein target may have been previously explored in the training data. Here, we implemented and evaluated several representative state-of-the-art methods, which covered a broad range of input formats and model architectures. More specifically, we considered typical inputs such as Morgan fingerprints, SMILES, graphs of compounds, and sequences of proteins. The tested model architectures contained multi-layer perceptrons (MLPs), convolutional neural networks (CNNs), graph neural networks (GNNs), and transformers. These methods included DeepCPI^40^, DeepDTA^41^, GraphDTA^42^, Tsubaki et al.^43^, DeepConvDTI^44^, MONN^37^, TransformerCPI^45^, and MolTrans^46^ (see “Methods” section for more details). The input formats and model architectures of these models are summarized in Supplementary Table 5.

**Fig. 3:**
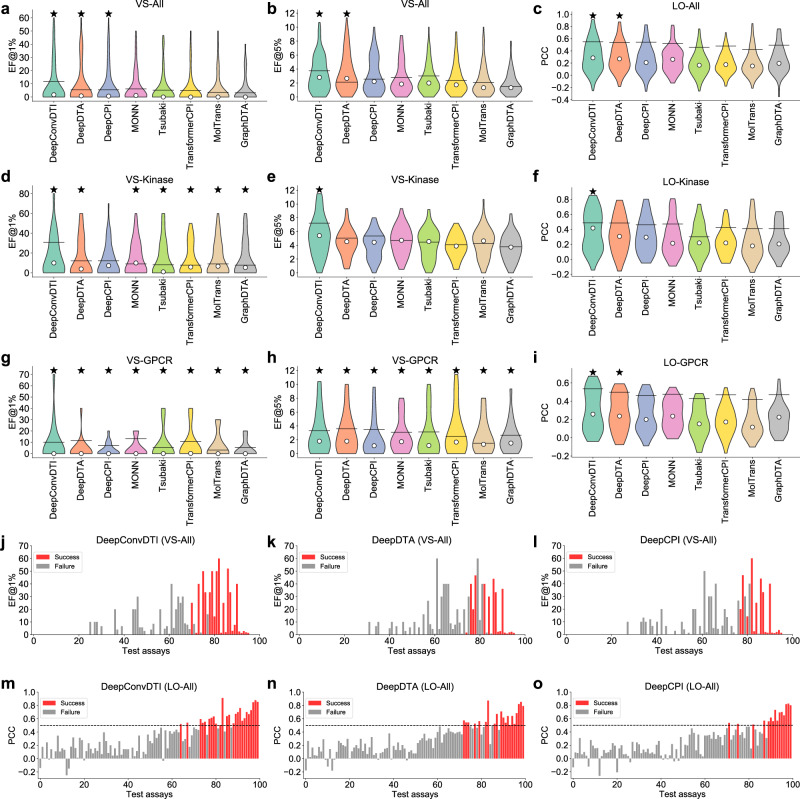
Performance evaluation of different compound activity prediction models under the zero-shot scenario. **a**, **b** Violin plots of per-assay enrichment factors at top 1% (EFs@1%, **a**) and enrichment factors at top 5% (EFs@5%, **b**) on the test assays of the VS-All task. **c** Violin plots of per-assay Pearson’s correlation coefficients (PCCs) on the test assays of the LO-All task. **d**, **e** Violin plots of per-assay EFs@1% (**d**) and EFs@5% (**e**) on the test assays of the VS-Kinase task. **f** Violin plots of per-assay PCCs on the test assays of the LO-Kinase task. **g**, **h** Violin plots of per-assay EFs@1% (**g**) and EFs@5% (**h**) on the test assays of the VS-GPCR task. **i** Violin plots of per-assay PCCs on the test assays of the LO-GPCR task. **j**–**o** Histograms of per-assay EFs@1% and PCCs achieved by DeepConvDTI, DeepDTA, and DeepCPI on the test assays of the VS-All (**j**–**l**) and the LO-All tasks (**m**–**o**). The success assays are colored in red. The dashed horizontal line stands for the threshold of 0.5 used for defining the success assays. The success rates (SRs) were defined as the portion of the success assays. In **a**–**i**, the best models and those with no significant difference compared to the best ones are marked with stars. The significance levels were calculated using two-sided *t*-tests adjusted by a false discovery rate of 0.05.

Through quantitatively evaluating the performances of different prediction models under the zero-shot scenario, the per-assay evaluation metrics are illustrated using violin plots for all six tasks of CARA (Fig. 3, Supplementary Fig. 2, and Supplementary Table 6). We observed that there existed gaps between the median per-assay metrics (white circles in the violin plots) and the all-assay metrics (black short horizontal lines) from the bulk evaluation (Fig. 3a–i), further suggesting the inaccurate estimation of bulk evaluation. Therefore, using all-assay metrics can be misleading when evaluating different computational models. We also found that the per-assay metrics can distribute in a relatively wide range among different assays even for the same model (Fig. 3j–o). For example, among 100 test assays of the LO-All task, the activity prediction models achieved the PCCs ranging from − 0.4 to 1.0 (Fig. 3c), and further inspection demonstrated that a compound activity prediction model may perform well in some cases while poorly in the others (Fig. 3m–o). As a result, evaluating models with a distribution of per-assay metrics over test assays instead of merely an averaged score can provide a more comprehensive assessment.

Although per-assay evaluation metrics can characterize the distributions of model performances, we still need one metric for directly ranking the prediction abilities of different models. Therefore, we examined the success rates of individual models, as defined in Section 2.2. The best success rate of the VS-All task was 39.40% while that of the LO-All task was 26.60%, both achieved by DeepConvDTI^44^ (Fig. 3 and Supplementary Table 6). Among all the tested models, five of them achieved over 30% success rates in the VS-All task, and two of them achieved over 20% success rates in the LO-All task. In summary, the assay-level evaluation of compound activity prediction models can provide a more comprehensive estimation of model performances, and thus narrow down the gap between in silico evaluation and real-world applications.

Noted that the VS-Kinase (VS-GPCR) and LO-Kinase (LO-GPCR) tasks were subsets of VS-All and LO-All tasks, respectively. To examine whether employing more training data, even from different target types, can improve the performances of compound activity prediction models in VS-Kinase (VS-GPCR) and LO-Kinase (LO-GPCR) tasks, we evaluated the test assays in these tasks using the models trained on all the training assays of VS-All or LO-All tasks. During dataset preparation, it was guaranteed that no overlapping samples existed between training and test assays from any tasks of All, Kinase, or GPCR. Therefore, there was no risk of data leakage when training on the VS-All (LO-All) task and evaluating on VS-Kinase (LO-Kinase) or VS-GPCR (LO-GPCR) tasks.

Direct comparison illustrated that training with more data (i.e., VS-All or LO-All) can improve the best success rates in the LO-Kinase and LO-GPCR tasks, while no significant influence on the best models was observed in the VS-Kinase and VS-GPCR tasks (Supplementary Table 7 and Supplementary Fig. 3). This result suggested that the VS-All and LO-All data in CARA were sufficient for training and evaluating the prediction models for various types of targets. We also observed that more training data cannot always lead to better performances (Supplementary Table 7 and Supplementary Fig. 3). Therefore we may assume that for a specific test assay, not all the training data were making a positive contribution to the prediction. To examine this assumption, we selected 20% of the training samples with the most similar compounds to train a specific model for each test assay. We also selected 20% of the training samples randomly from the original training data for a fair comparison. We trained DeepConvDTI, one of the best compound activity prediction methods according to Supplementary Table 6, in both VS-All and LO-All tasks. The models trained on 20% similar samples significantly outperformed those on 20% random samples in both VS-All and LO-All tasks (Supplementary Fig. 4). We also observed that the model trained on 20% of most similar training samples achieved comparable performances to that trained on all data (Supplementary Fig. 4). These results indicated that a small portion of the training samples may be sufficient to provide informative information for training for an individual test assay. As a result, we can train a compound activity prediction model with part of the training samples (e.g., 20%) that are most similar to the compounds to be predicted to save computational resources in practical applications. On the other hand, as the non-similar samples may not decrease the performance, we can also train a model safely using the whole training dataset once for all test assays under the zero-shot scenario.

### Performance evaluation of models and training strategies under the few-shot scenario

The above zero-shot scenario focused on the scenario when there was no labeled data for a specific application in drug discovery. It is also common to face a situation in which the candidate compounds need to be further screened or optimized according to a small number of existing compounds with already measured or reported activity data. Here we formulated the activity prediction problems in the latter situation as the few-shot learning scenario^38^, in which the models are required to predict compound activities for specific assays with a small amount of labeled data (i.e., randomly chosen 50 compounds from each test assay).

**Fig. 4:**
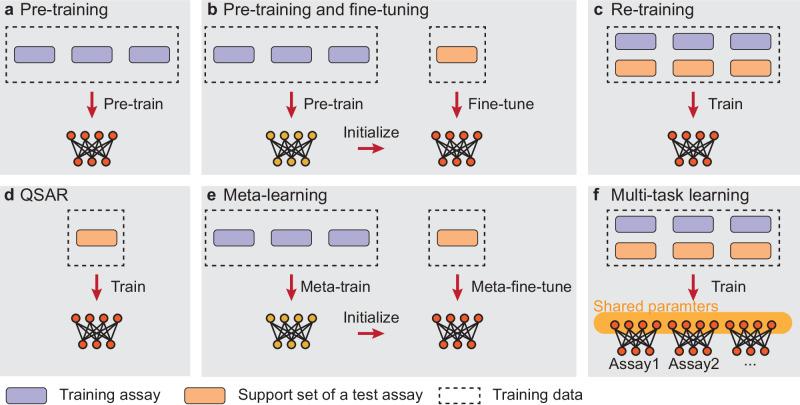
Illustration of different training strategies evaluated under the few-shot scenario on the CARA benchmark. **a** Pre-training (i.e., zero-shot learning). **b** Pre-training and fine-tuning. **c** Re-training. **d** Quantitative structure-activity relationship (QSAR). **e** Meta-learning. **f** Multi-task learning. Refer to the main text for more details about these training strategies.

In the CARA benchmark, we also evaluated several classical few-shot learning strategies for developing machine learning and deep learning models in the few-shot scenario (Fig. 4 and “Methods” section):

#### Quantitative structure-activity relationship

A QSAR^28^ model tries to capture the relationships between the structures and activities mainly based on compound structures. Since the protein information is not considered in the QSAR models, they were trained for individual assays separately. Here, we trained a QSAR model for each test assay using the compounds in the support set only. We implemented typical QSAR algorithms including random forest (RF)^47^, gradient boosting tree (GBT)^48^, support vector machine (SVM)^49^, and deep neural network (DNN)^50^.

#### Pre-training and fine-tuning

Pre-training on large-scale data is believed to be able to learn useful feature representations and thus improve the performances on specific tasks after fine-tuning^51,52^. In this study, a model was first pre-trained on all the training assays and then fine-tuned on the support set of each test assay individually. Note that the trained models in the zero-shot scenario can also serve as the pre-trained models here. Thus we directly adopted these models obtained from the zero-shot scenario to save computational resources. Here, the top three best models, i.e., DeepConvDTI^44^, DeepDTA^41^, and DeepCPI^40^, derived from the zero-shot scenario were tested.

#### Meta-learning

We adopted the popular model-agnostic meta-learning (MAML) framework^26^ as our meta-learning strategy tested in this study. More specifically, the model was first meta-trained on all the training assays and then meta-fine-tuned on the support set of each test assay individually. We implemented the meta-learning strategy for three models, i.e., DeepCPI-c, MTDNN, and DeepConvDTI-c, corresponding to ones derived using the compound encoders and activity prediction modules from DeepCPI^40^, MTDNN^53^, and DeepConvDTI^44^, respectively (see “Methods” section).

#### Multi-task learning

The multi-task learning^27^ model can be benefited from the shared information among different tasks through training these tasks together. A multi-task learning model generally consists of both shared and task-specific parameters. Here the shared parameters were trained using the samples from the same training assays as in meta-training, plus the support sets of all test assays. The task-specific parameters were only updated based on the samples of the corresponding training or support assays. We employed the MTDNN^53^ model which was originally designed for solving the multi-task learning problem in kinase inhibitor activity prediction. We also implemented another model for the multi-task learning strategy, named DeepCovDTI-c, which was constructed using a compound feature encoder from DeepConvDTI^44^ and a similar multi-task feature decoder in MTDNN^53^ (see “Methods” section for more details).

#### Re-training

A re-training model was trained using the training assays and support sets of all the test assays as in multi-task learning. Since the re-training models should incorporate the information from different assays, models that took both protein and compound features as inputs can be applied. We thus evaluated DeepConvDTI^44^, DeepDTA^41^, and DeepCPI^40^ for the re-training strategies here.

**Fig. 5:**
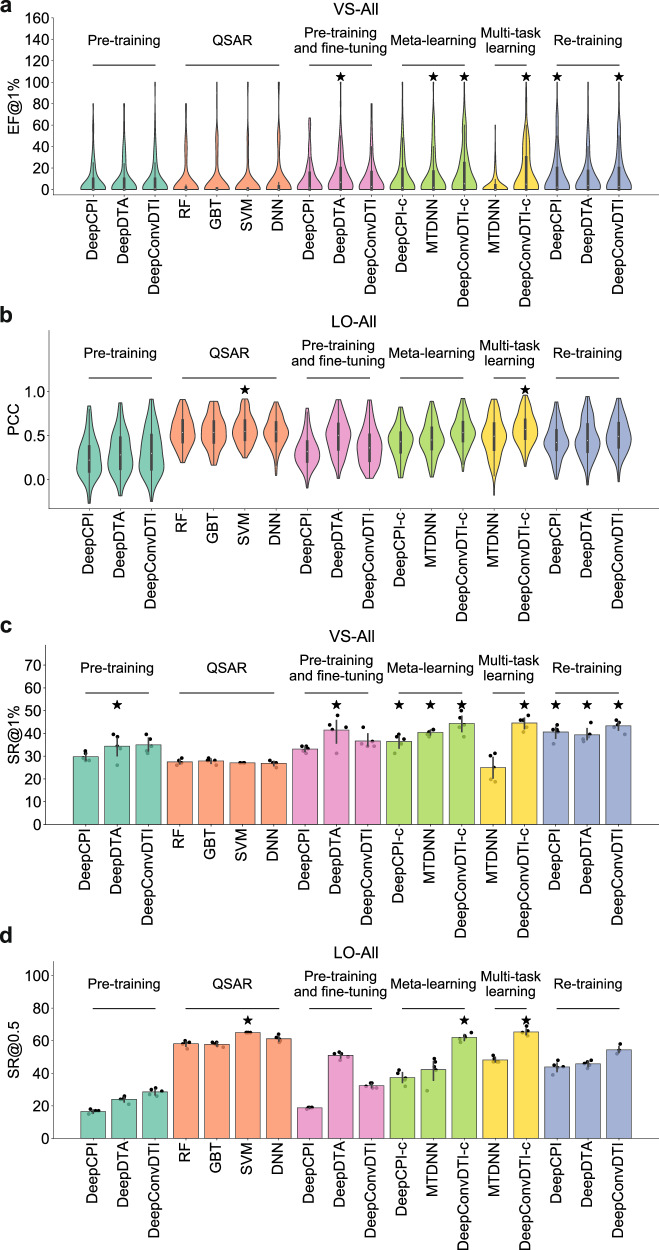
Performance evaluation of different training strategies under the few-shot scenario. **a** Violin plots of per-assay enrichment factors at top 1% (EFs@1%) for different training strategies on the test assays of the VS-All task. **b** Violin plots of per-assay Pearson’s correlation coefficients (PCCs) for different training strategies on the test assays of the LO-All task. **c** Box plots of per-assay success rates at top 1% (SRs@1%) for different training strategies on the test assays of the VS-All task. **d** Box plots of per-assay success rates with PCC > 0.5 (SRs@0.5) for different training strategies on the test assays of the LO-All task. The best strategies and those with no significant differences compared to the best ones are marked with stars. The significance levels were calculated using two-sided *t*-tests adjusted by a false discovery rate of 0.05.

Compared to the zero-shot scenario (i.e., pre-training), the success rates of the best model in the few-shot scenario were improved by up to 9.80% and 36.80% for the VS-All and LO-All tasks, respectively (Fig. 3a–c, Supplementary Fig. 2a, j, Fig. 5, and Supplementary Tables 6 and 8). The average per-assay EF@1% for the VS-All task and average PCC for the LO task were also significantly increased by 6.78 and 0.26, respectively. Such improvements were possibly benefited from the assay-specific structure-activity relationship information provided by the support sets of test assays. As a result, providing a small number of assay-related labeled data (50 support samples here) can introduce substantial improvement in the success rates of compound activity prediction.

We also noticed that different training strategies in the few-shot scenario exhibited distinct performances. For the VS-All task, pre-training and fine-tuning, meta-learning, multi-task learning, and re-training strategies were significantly better than the QSAR models (Fig. 5a, c and Supplementary Table 8), indicating that the cross-assay information from the large-scale training data can help boost the prediction performances on individual VS assays. For the LO-All task, however, the best QSAR model achieved similar performances to the best meta-learning and multi-task learning models. These three strategies, i.e., QSAR, meta-learning, and multi-task learning, all achieved success rates of about 65%, without statistically significant difference (Fig. 5b, d and Supplementary Table 8). As the QSAR models were trained on a much smaller dataset (i.e., only the support set) than the other strategies, the above result may suggest that additional information from the other assays may not contribute much to the prediction for the assay of interest in the LO tasks.

For the few-shot scenario, the number of few-shot samples (i.e., the size of the support set) is critical to the performances of computational models and training strategies (Supplementary Fig. 5a, b). For both VS-All and LO-All tasks, the performances were improved with the increase of support samples for all three strategies considered in this analysis, including QSAR, pre-training and fine-tuning, and meta-learning. Interestingly, we observed that in the VS-All task, the success rate of the QSAR model trained with 50 samples was not better than those of the other two strategies even with 10 few-shot samples (Supplementary Fig. 5a). Noted that pre-training and fine-tuning as well as meta-learning strategies both contained a pre-training or meta-pre-training process, which suggested that employing more training data was helpful for the VS task. On the other hand, these two strategies with pre-training failed to outperform the QSAR model in the LO-All task (Supplementary Fig. 5b). This result further indicated that the training samples may not provide more useful information than the support samples for the LO task.

To further understand the model behaviors related to the fine-tuning steps in different training strategies, we also visualized their learning curves (Supplementary Fig. 5c, d). We used three models as the starting points of fine-tuning, including a randomly initialized model, a pre-trained model, and a meta-trained model. In both VS-All and LO-All tasks, we observed that the meta-trained model converged quickly after several steps, while the randomly initialized model and the pre-trained model required more steps to reach stable performances. This observation was in accordance with the goal of meta-learning, that is, to find a proper initial model that can quickly achieve good performances after a few updates with the support sets^26^. Before fine-tuning on the assay-specific data (i.e., when the number of fine-tuning steps was equal to zero), the pre-trained models performed better than the randomly initialized models in both VS-All and LO-All tasks, as well as the meta-trained model in the LO-All task. However, the performances of the pre-trained model decreased in the beginning steps of fine-tuning and then increased slowly. This may be possibly explained by the fact that the pre-training process aimed to obtain the best-fitted model for the training data, and thus the pre-trained model might be over-fitted to the training data and thus need a certain number of steps to escape from the local optima.

In summary, the performances in activity prediction in the few-shot scenario depended on the selection of models, training strategies, and task types (i.e., VS or LO). All these variables should be considered when developing the few-shot learning models in practical applications.

### Influence of data distributions on selecting the best training strategies

**Fig. 6:**
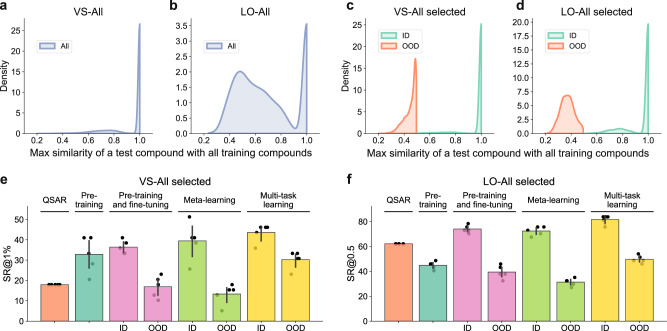
Relationships between the distributions of training samples and the performances of different training strategies under the few-shot scenario. **a**, **b** Distributions of the maximum similarity scores of test compounds compared with training data in the VS-All (**a**) and LO-All (**b**) tasks, respectively. The maximum similarity score was defined as the maximum Jaccard similarity between the molecular fingerprints of each test compound and all the training compounds. **c**, **d** Distributions of the maximum similarity scores of test compounds compared with training data in the selected test assays, in which both in-distribution (ID) and out-of-distribution (OOD) training data were available, in the VS-All (**c**) and LO-All (**d**) tasks, respectively. The similarity score was defined as the maximum Jaccard similarity between molecular fingerprints of each test compound and the corresponding ID or OOD subsets of training compounds. **e**, **f** The success rates of the best models (i.e., SVM for QSAR, DeepDTA for pre-training, DeepDTA for pre-training and fine-tuning, DeepConvDTI-c for meta-learning and multi-task learning) achieved using different training strategies and training data (i.e., ID or OOD) on the selected test assays for the VS-All (**e**) and LO-All (**f**) tasks, respectively. The success rates at top 1% (SRs@1%) were used for the VS-All task while the success rates with PCC > 0.5 (SRs@0.5) were used for the LO-All task. Refer to “Methods” section for more details about the selection of ID or OOD training assays and the test assays. The error bars in **e** and **f** indicate the standard deviations over five repeats.

In the above section, we observed that the cross-assay information employed in re-training, pre-training and fine-tuning, meta-learning, and multi-task learning strategies significantly boosted the prediction performances in the VS tasks, compared with the QSAR models without using data from the training assays. However, the contribution of the cross-assay information was almost negligible in the LO tasks. To explore the underlying reasons for such differences between VS and LO tasks, we first visualized the data distributions in the two tasks (Fig. 6a, b). For the VS-All task, although we adopted the new-protein splitting scheme to ensure that the training and test proteins did not overlap, the compounds exhibited relatively high similarity between training and test assays (Fig. 6a). More specifically, there existed 79% of test compounds for which the corresponding training compounds had similarities over 0.9. In contrast, for the LO-All task, the compound similarities between training and test assays were relatively low (Fig. 6b). The percentage of test compounds with similarities to the corresponding training compounds over 0.9 was only 27%.

Based on the above observations, we hypothesized that the compound space of the VS-All task was shared between training and test assays. In other words, the compounds in the test assays were in-distribution (ID) with those in the training assays (Fig. 6a). Therefore, the models can learn useful information from the training assays to boost their prediction performances on the test assays. In contrast, for the LO-All task, the compound distributions of the training and test assays were not shared, or out-of-distribution (OOD) (Fig. 6b). Thus, learning from the training assays can hardly improve the prediction performances on the test assays.

To examine the above hypothesis that the ID/OOD distribution of training and test compounds may affect the selection of the best training strategies, we specially designed the VS and LO tasks with a set of selected assays containing either ID or OOD training data (more details can be found in Methods). The constructed ID datasets of both VS and LO tasks exhibited high similarities between training and test compounds (Fig. 6c), while such similarities were low in the constructed OOD datasets (Fig. 6d). For a fair comparison, the selected test assays and the sizes of training data were exactly the same between ID and OOD datasets (Supplementary Table 9). Two strategies employing the cross-assay information, including pre-training and fine-tuning, and meta-learning, were evaluated. In particular, the models were first pre-trained or meta-trained on the ID or OOD training datasets, and then fine-tuned or meta-fine-tuned on the support sets of the selected test assays individually.

We observed that the fine-tuned, meta-fine-tuned, and multi-task learning models achieved significantly better performances than the QSAR models on the constructed ID datasets of both VS and LO tasks (Fig. 6e, f). These results suggested that employing the cross-assay information from the ID training data can be helpful compared to the case using only the support set of test assays. In contrast, those models trained on the OOD data achieved relatively lower performances, even worse than the QSAR models. These results further supported the hypothesis that the OOD samples from training assays were not beneficial for making predictions for test assays. The reason that the VS tasks benefited more from the strategies exploring the cross-assay information may be because the compounds in the test data were partly in-distribution with training compounds. For the LO tasks in which most samples in the test set were out-of-distribution with training data, the information in training samples was limited for achieving better performance. Therefore, the data distribution can provide a useful guideline for choosing the best training strategies for the VS and LO tasks. In summary, when adopting the few-shot training strategies in practice, it is important to consider whether the ID training samples are available for specific applications. In other words, incorporating the activity data related to the compounds of interest may help build a more reliable model.

### Factors and indicators related to model confidence

In this section, we first analyzed the possible factors that can affect activity prediction performances to help understand the behaviors of models (Supplementary Fig. 6). We observed that in the VS-All task, the success rate of an individual assay reached almost 100% when the number of samples in that assay was larger than 10,000 (Supplementary Fig. 6b). This was probably because the number of hits in the virtual screening task by randomly selecting 1% of compounds without replacements followed a hypergeometric distribution, and thus the expectation of the hit number would be larger than one if the population size exceeded 10,000 (see “Methods” section for more details). As a result, the VS assays with a large number of candidate compounds (e.g., greater than 10,000) were expected to succeed (i.e., at least one hit compound in the top 1%) even by random guess. We also observed that the standard deviations (SDs) of labels were positively correlated with the model performances in the LO-All task (Supplementary Fig. 6g, h). This result indicated that an assay with a smaller activity range may be more difficult to distinguish for a prediction model. In addition, no direct conclusions can be made for different measurement types (Supplementary Fig. 6i–l) or target types (Supplementary Fig. 6m–p) probably due to the limited number of assays for each measurement type or target type. We also considered the pharmacological profiles measured by different assays, such as agonism, antagonism and binding (Supplementary Data 1). We summarized the pharmacological profiles of the test assays in CARA and observed that the types of pharmacological profiles did not significantly influence the model performance, according to the evaluation on the VS-GPCR and LO-GPCR datasets (Supplementary Note 2, Supplementary Figs. 13, 14, and Supplementary Table 10). However, we found that the extent of unexploration of the compounds in a test assay, defined according to their similarities with the training compounds, may influence the performances of activity prediction models. Those test assays with more unexplored compounds (i.e., those with lower similarities with training compounds) tended to have relatively worse performances, although our few-shot training strategies were still effective on such assays (Supplementary Note 3, Supplementary Fig. 15, and Supplementary Table 11).

**Fig. 7:**
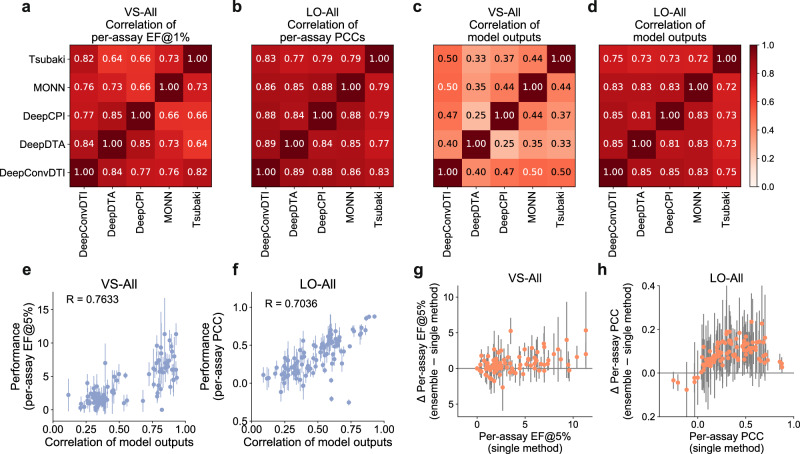
Indicators correlated with model performances. **a** The correlations of per-assay EFs@1% between two models on all the test assays for the VS-All task. **b** The correlations of per-assay PCCs between two models on all the test assays for the LO-All task. **c**, **d** The correlations of model outputs (i.e., the predicted activities of trained models on the samples of individual test assays.) between two models on all the test assays for the VS-All (**c**) and LO-All (**d**) tasks, respectively. **e**, **f** The correlations of model outputs were correlated with model performances in both the VS-All (**e**) and LO-All (**f**) tasks, and thus can serve as a good indicator of model confidence. **g**, **h** The increase in performance was achieved by an ensemble of the five best methods (i.e., DeepConvDTI, DeepDTA, DeepCPI, MONN, and Tsubaki et al.), compared to single methods in the VS-All (**g**) and LO-All (**h**) tasks, respectively. The average increases in performance over the five best methods for each assay are shown. The error bars refer to the standard deviations over the five best methods.

In practice, it would be desirable to estimate the confidence of the model outputs before knowing the real activities of candidate compounds. To achieve this goal, we investigated the relationships between the model performances and several potential indicators that can be observed from the model outputs only. More specifically, we first analyzed the correlation between the performances of different compound activity prediction methods. Through computing the correlations between per-assay EFs@1% or PCCs of any two methods (Fig. 7a, b), we found that the performances of those compound activity prediction methods were highly correlated in both VS and LO tasks. This suggested that the methods with distinct architectures can achieve similar performances on individual assays. In addition, positive correlations were also observed between the outputs of different methods (Fig. 7c, d), suggesting that different methods demonstrated certain levels of accordance in their prediction outputs. We further inspected the correlations of model outputs at the assay level and observed that the output correlations varied among different assays. For assays with higher output correlations, their performances were generally better. In contrast, for those assays with lower output correlations, the corresponding performances were quite poorer (Fig. 7e, f). The inaccurate prediction results of an assay may associate with different kinds of errors by different models, and therefore there existed no correlation among their outputs. As a result, the output correlation between different methods can serve as a useful indicator to estimate the prediction performances of the methods on individual assays, and thus guide the applications of these methods in practice. Apart from the output correlations between different methods, we observed that the correlations of model outputs among different repeats of the same method also showed good correlations with the model performances (Supplementary Fig. 7).

The above observations of the assay-level accordance in different methods further suggested that it is possible to ensemble several methods to gain better prediction performance. Since significant correlations in model outputs were only observed in the correctly predicted test assays, such an ensemble strategy was expected to boost the performances of these assays. As expected, the ensemble models did improve the prediction results of those assays in which the single methods demonstrated certain prediction ability (e.g., those assays with single-model performances of PCC > 0.3 in the LO-All task, Fig. 7g, h).

### A practical pipeline for choosing the useful prediction methods under different application scenarios

**Fig. 8:**
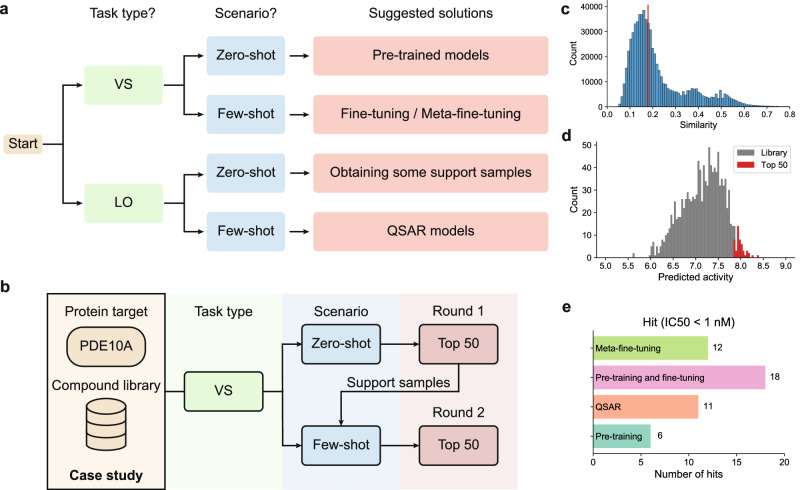
Schematic illustration of the proposed solutions and a case study on the practical applications of compound activity prediction methods, based on the observations derived from CARA. **a** A proposed pipeline for suggesting useful training strategies for practical compound activity prediction. **b** The overall screening process of the case study related to PDE10A. Two rounds of screening were conducted corresponding to the zero-shot and few-shot scenarios, respectively. **c** Histogram of pairwise similarities of the 1162 compounds in the case study. The median pairwise similarity is shown with the red line. **d** The predicted activities of 1162 compounds using the pre-trained models in the first round, including DeepConvDTI, DeepDTA, and DeepCPI. For each compound activity prediction method, we averaged the predicted activities over five repeats. Then, these predicted activities were averaged over three methods as the final prediction scores for all the compounds. The top 50 compounds with the highest predicted activities were highlighted in red. **e** Numbers of hits from the top 50 compounds scored by different strategies in the second round, under the threshold of 1 nM. For each strategy, the prediction scores of several models (DeepConvDTI, DeepDTA, and DeepCPI for pre-training and fine-tuning, DeepConvDTI-c and DeepCPI-c for meta-fine-tuning and RF, GBT, and SVM for QSAR) and five repeats were averaged.

According to the comparison results of different methods and training strategies on VS/LO tasks under the zero-shot/few-shot scenarios, we proposed a useful pipeline for the practical drug discovery applications, as shown in Fig. 8a. The pipeline identifies practical solutions for individual drug discovery projects according to the corresponding task types and data settings (Fig. 8a). For the VS tasks, we recommend existing deep learning-based pre-training models under the zero-shot scenarios and their fine-tuned or meta-fine-tuned version under the few-shot scenarios, respectively, as they exhibited a relatively higher level of success rates on such tasks in our previous analyses (Figs. 3 and 5). For the LO tasks, since current computational methods exhibited relatively limited ability under the corresponding zero-shot scenario (Fig. 3), we suggest to first obtain the activity labels for some of the compounds through experimental assays to convert the zero-shot problem into a few-shot one. Then, the QSAR models, which achieved the best performance on the LO tasks in the few-shot scenario (Fig. 5), can be applied to make predictions for the rest compounds.

We employed a case study to demonstrate a simulated compound screening process using our proposed pipeline. In particular, the cAMP and cAMP-inhibited cGMP 3^′^,5^′^-cyclic phosphodiesterase 10A (PDE10A) is an enzyme in human that is associated with hyperkinetic movement disorder. Recently, Tosstorff et al. released an industrial dataset containing 1162 chemical compounds used for screening the PDE10A inhibitors^54^. Through analyzing the molecular similarities between these compounds and those in the training data of our CARA benchmark, we found that 1088 (94%) compounds in this new dataset were unexplored chemical compounds with maximal similarities < 0.5. Therefore, we could use this set of compounds as a practical example of VS tasks for novel drug discovery.

A two-round screening process based on this dataset is described in Fig. 8b. First, we computed the pairwise similarity scores of the compounds in this dataset, and obtained a median pairwise similarity of 0.18 (Fig. 8c). According to the original threshold for distinguishing VS and LO tasks (i.e., the median pairwise similarity score of 0.2), the case analyzed here was in the scope of VS tasks. Because there existed no labeled samples before conducting experiments, we can only choose zero-shot as the training scenario in the first round. According to the solution suggested by CARA (Fig. 8a), we predicted the compound activities under the zero-shot scenario for this VS task using the pre-trained models. The predicted activity values of all the 1162 compounds against the protein target PDE10A are shown in Fig. 8d. To evaluate the predictive power of these pre-trained models, we selected the top 50 compounds with the highest prediction scores for validation. Among these top 50 predicted compounds, seven of them exhibited the IC_50_ values better than 1 nM, that is, a hit rate of 14% among the top 50 predictions. In comparison, the overall hit rate in this dataset was only 6.5% (74 out of all the 1162 compounds), thus indicating the effectiveness of the pre-trained models in virtual drug screening.

In practice, the drug discovery process usually undergoes several cycles of screening or optimization. Benefiting from the above 50 compounds with labeled activities, we were then able to conduct a second round of screening. In this stage, the task type of screening the remaining 1,112 compounds still belonged to the VS task, but the training scenario was changed into the few-shot scenario. According to the solutions suggested by CARA from Fig. 8a, we can fine-tune or meta-fine-tune the models using the previously labeled compounds. Here, we conducted both fine-tuning and meta-fine-tuning strategies for evaluation, and also included the pre-training and QSAR approaches for comparison. More specifically, we trained or fine-tuned the few-shot models using the activity labels of the top 50 compounds identified from the first round, and then for each strategy, we selected another 50 compounds with the highest prediction scores from the remaining 1112 compounds for validation. As shown in Fig. 8e, for the two suggested strategies, i.e., fine-tuning and meta-fine-tuning, the numbers of hits were relatively higher, compared to the other two strategies. Of the top 50 compounds suggested by each strategy, fine-tuning highlighted 18 compounds with high potency that exhibited the IC_50_ values better than 1 nM. The numbers of hits for meta-fine-tuning, QSAR, and pre-training were 12, 11, and 6, respectively. In other words, the best hit rate achieved by the suggested strategies outperformed the others by 64–200%.

Taken together, the above results suggested that the conclusions and insights obtained by CARA may greatly facilitate the applications of machine-learning models in the drug discovery process, through using different models and training strategies according to specific conditions.

### Challenges in making predictions related to activity cliffs

The concept of activity cliff (AC) describes a commonly observed phenomenon in drug design in which a pair of structurally similar compounds can sometimes exhibit totally distinct activities^55^. In other words, a minor change in the local structure may cause a large drop in the activity space like a cliff. The frequent occurrences of activity cliffs imply a complicated and non-smooth landscape about the relationships between structures and activities in the chemical spaces of compounds^56^. Many examples of activity cliffs were previously reported in medicinal chemistry studies, and they were often critical to drug design but not easy to be predicted or interpreted^57^. A recent study showed that a higher proportion of activity cliffs in the datasets can lead to poorer prediction performances of the QSAR models^58^. Here, we examined the effects of activity cliffs on the prediction performances of different computational approaches on our CARA benchmark.

Similar compounds can be defined by several criteria including fingerprint similarity, matched molecular pairs (MMPs), and analog pairs^59^. The large activity discrepancy was often determined by at least ten or 100 times difference in activity values, that is, absolute difference larger than one or two in p(activity) values^59^. In this work, we followed the previous studies of defining activity cliffs using MMPs, which described pairs of compounds with a small change in their chemical structures^60^. In particular, a pair of activity-cliff compounds (termed AC pairs) was defined as an MMP with an absolute difference > 1 in their p(activity) values. We analyzed the 100 test assays of the LO-All task and there were 15% AC pairs among all the MMP pairs (Supplementary Fig. 8a). The VS tasks were not included in this analysis, as they generally consist of compounds with relatively low similarity, and activity cliff was a relatively less important issue in the drug screening stage. To further describe whether a compound was frequently involved in activity cliffs (i.e., to what extent the activity landscape near this compound was discontinuous), we extended the discontinuity score proposed in^61^ to the analyses of MMP-defined activity cliffs. More specifically, the discontinuity score was defined as the weighted average of activity differences between a specific compound and its MMP neighbors (Methods). According to this definition, we termed the activity-cliff compounds (AC compounds) as those with discontinuity scores > 1, which accounted for about 6% of compounds in the test assays of the LO-All task (Supplementary Fig. 8b). Examples of test assays with AC pairs and AC compounds are demonstrated in Supplementary Fig. 8c.

We next compared the sample-level performances of the cliff prediction task using the mean absolute error (MAE) of AC and non-AC compounds on the test assays of the LO-All task (Methods). The cliff predictions (i.e., activity differences) were derived from the best compound activity prediction model in the few-shot scenario, i.e., DeepConvDTI-c from multi-task learning. As a result, among the 18 assays containing more than 20 AC compounds, we found that there were 14 assays where the MAE scores of AC compounds were significantly larger than those of non-AC compounds (Supplementary Fig. 8d). This result indicated that the AC compounds generally represented more difficult cases for the compound activity prediction models. Further analysis implied that the differences in model outputs can be applied to classify AC pairs only when the activity prediction model was of high performances (Supplementary Fig. 8e). Therefore, identifying activity cliffs through compound activity prediction models is still a challenging problem.

## Discussion

In this paper, we proposed the CARA benchmark for evaluating and developing machine learning and deep learning-based computational methods as well as training strategies in predicting compound activities from a practical perspective of drug discovery. We curated and organized the assay-based experimental data into VS and LO tasks and evaluated these tasks in the few-shot or zero-shot scenarios, to cover various types of application situations with different purposes, resources, and data distributions. To better estimate the model behaviors in real-world activity prediction tasks, we designed the assay-based evaluation metrics, which can display more comprehensive and accurate results compared with the commonly used bulk evaluation metrics. Through testing typical types of few-shot strategies, we concluded that different training strategies should be preferred in distinct tasks. That is, those strategies through exploring the cross-assay information can be useful for the VS tasks, while the QSAR models through employing only single-task information were among the top-performing ones for the LO tasks. Such differences can be partially explained by the distributions of training and test compounds: those in-distribution training data were more informative for making predictions on the compounds in the test assays. Further analyses demonstrated that different models achieved certain levels of accordance in those assays with relatively high performances, thus providing a helpful indicator for estimating the reliability of model outputs before knowing the real activity labels. In addition, our analyses revealed that there still existed certain unsolved challenges for current compound activity prediction problems, such as activity cliff prediction.

It is also important to further investigate whether it is possible to estimate sample-level confidence of the prediction results, which is related to the concept of uncertainty estimation^62^. However, commonly used uncertainty metrics, including the standard deviation of outputs from different models and the variance estimated by Gaussian process regressors, though being successful in several applications^62^, can hardly capture the sample-level prediction errors in the tasks defined in our CARA benchmark (Supplementary Fig. 9). Therefore, it remains an open challenge for developing computational methods that can provide accurate sample-level confidence or uncertainty estimation for compound activity prediction tasks.

Although the structure-based methods are also a major group of computational activity prediction approaches, their performances were not evaluated on the CARA benchmark. One reason was that there were no experimentally-solved protein structures for most proteins in the ChEMBL database^14^. Therefore, we mainly focused on the sequence-based methods in the CARA benchmark, in which the protein features are encoded with their primary sequences. In addition, though currently it is possible to predict protein structures from primary sequences^63^, their binding pockets of the ligand compounds are still unknown. As most structure-based computational methods require the positions of protein pockets as inputs, it is not straightforward to assign appropriate protein structures with correct pockets for the massive assays in the CARA benchmark. Additional efforts should be made for narrowing down the gap between the sequence- and structure-based data, and we leave the comprehensively designed structure-based version of the CARA benchmark in our future work. Nevertheless, we still mapped about 20% of the proteins in the test assays of the VS-Kinase and LO-Kinase tasks to the proteins with known structures and pocket locations from the PDB database^64^ to enable the comparison between the sequence-based methods and a number of widely-used molecular docking-based approaches (Methods). On these subsets of the test assays, the sequence-based deep learning-based methods generally achieved significantly better performances than three popularly used docking-based methods including AutoDock Vina^65^, iDock^66^, and LeDock^67^ (Supplementary Fig. 10). These results indicated that the data-driven methods, although trained in a zero-shot scenario and with the new-protein splitting scheme using only protein sequences as inputs, demonstrated greater power in predicting compound activities for the drug screening tasks.

Although the CARA benchmark provided a comprehensive evaluation on the capability of existing computational methods, it did not aim to solve all the problems related to compound activity prediction in drug discovery. For example, drug repurposing is another popular strategy in drug discovery and development, which aims to discover new relations between existing drugs and targets^68^. There are generally relatively smaller numbers but richer information for those investigated drugs and targets, and thus the problem formulation, the design of computational models, and evaluation metrics used in drug repurposing tasks can be quite different from those used in current CARA benchmark^69^. Selectivity is another important problem in drug design, which characterizes the activities of the same compound against structurally or functionally similar protein targets^17^. The current version of CARA was not directly suitable for evaluating the selectivity prediction problem, since there were only a few similar proteins in the test assays. Selecting appropriate benchmark datasets according to the specific problems encountered in the drug discovery process is important for obtaining precise evaluations of the corresponding computational methods. Therefore, more efforts in developing task-specific benchmark datasets will be needed in the future.

## Methods

### Construction of the CARA benchmark

#### Data filtering and organization

The CARA benchmark dataset was collected from the ChEMBL database^14^ (version 30). The samples (i.e., compound-protein pairs with activity values) in the ChEMBL database were filtered according to the following criteria: First, only samples with the target type SINGLE PROTEIN were kept. Samples with multiple protein sequences were removed to avoid ambiguity. Second, those non-small-molecule ligands with molecular weights larger than 1000 were removed. Third, we only kept those well-annotated samples with no missing values in assays.chembl_id, target_dictionary.chembl_id, component_sequences.sequence, component_class.protein_class_id, molecule_dictionary.chembl_id,compound_structures.canonical_smiles, and activities.pchembl_value. In addition, the activity values measured in terms of inhibition rates or activation rates were removed. Those assays containing only one unique sample were also excluded.

After filtering, all the samples were first organized into individual ChEMBL assays according to their assays.chembl_id values. Then, a small number of ChEMBL assays containing multiple measurement types (e.g., K_i_, K_d_, IC_50_, EC_50_, and potency) were further separated, so that each assay defined in CARA contained only one measurement type. Here, we combined assays.chembl_id values with the measurement types as the assay IDs in CARA (e.g., CHEMBL4426324_Ki). The activity labels of replicates within the same assay were merged and their median values were used as the final labels.

#### Assay categorization

Based on the compound similarity patterns in individual assays, we categorized them into virtual screening (VS) or lead optimization (LO) assays. For VS assays, diverse compounds are generally screened to discover active ones, while for LO assays, compound series containing structurally similar compounds are often tested. The similarity between the two compounds was calculated using the Tanimoto similarity of their Morgan fingerprints. For each assay, the median value of all the pairwise compound similarities was calculated. According to the distribution of median similarities of all assays (Supplementary Fig. 11), the VS and LO assays could be distinguished using an empirical threshold of 0.2. That is, the assays with median pairwise compound similarities no greater than 0.2 were assigned as the VS assays, while those larger than 0.2 were assigned as the LO assays.

#### Data splitting schemes

All the assays were split into training and test assays for model training and evaluation, respectively. To systematically evaluate different compound activity prediction models, representative assays with diverse protein targets were first selected as test assays. More specifically, the assays were first clustered according to their protein similarities, which were calculated by normalizing their sequence alignment scores derived from the Smith-Waterman algorithm as in a previous study^37^. Hierarchical clustering with a similarity threshold of 0.4 was adopted. That is, the similarity between any two proteins from different clusters must be smaller than 0.4. Then, 20% and a maximum of 100 protein clusters were selected as the test clusters for each CARA task. Within each test cluster, the assays with abundant samples (i.e., at least 100 samples), proper label range (i.e., the difference between the maximal and minimal activities greater than 2 in p(activity)), and diverse label values (i.e., the number of unique activity values greater than 10) were assigned as the test assays. Those assays that were not in the test clusters were assigned as the training assays for both VS and LO tasks. In addition, for the VS tasks, the remaining assays in the test clusters were discarded, resulting in a new protein-splitting scheme. For the LO tasks, the remaining assays in the test clusters were assigned as training assays, resulting in a new-assay splitting scheme. Finally, all the compound-protein pairs that appeared in the test assays were removed from the training assays to avoid data leakage.

The data splitting schemes were adopted in both zero-shot and few-shot scenarios. Additionally, in the few-shot scenario, 50 samples in each test assay were randomly selected as the support set and the remaining samples were used as the query set. Since each test assay was ensured to contain at least 100 samples, the size of the query set was always equal to or greater than 50.

### Compound activity prediction methods

Several representative compound activity prediction methods were used for evaluation in this study. DeepCPI^40^ utilized a two-stage strategy for predicting compound activities. Compound and protein features were first extracted and compressed using singular value decomposition (SVD) based on the Morgan fingerprints of compounds and a 3-mer encoding feature encoding scheme of proteins, respectively. Then, fully connected layers taking the concatenated features of compounds and proteins as inputs were trained to predict their interactions. DeepDTA^41^, in contrast, predicted the compound activities in an end-to-end manner. The features of input molecules were learned through convolutional neural networks (CNNs) from the SMILES (simplified molecular-input line-entry system) strings of compounds and the primary sequences of proteins. GraphDTA^42^ improved DeepDTA through utilizing graph neural networks (GNNs) to encode the molecular graphs of compounds, in which atoms and bonds of a compound were considered as nodes and edges in a graph, respectively. Tsubaki et al.^43^ introduced another compound protein interaction prediction approach using GNNs and CNNs to learn the features of compounds and proteins, respectively. DeepConvDTI^44^ also predicted the compound activities with a CNN-based architecture for encoding the protein features, but the compound features were represented by Morgan fingerprints and encoded by fully connected layers. MONN^37^ further introduced a graph warp module for encoding compound features and dual memory attention in its activity prediction module. TransformerCPI^45^ employed a transformer decoder architecture to predict compound activities from the input compound SMILES strings and protein sequences. MolTrans^46^ introduced an augmented transformer encoder to learn the sub-structural patterns of compound activities.

In addition, MTDNN^53^ proposed a multi-task neural network to learn the activities of inhibitors against different kinases. The commonly used QSAR models, which predict the activity values only based on compound information (typically fingerprints) and machine learning methods, such as random forest (RF)^47^, support vector machine (SVM)^49^, gradient boosting tree (GBT)^48^, and deep neural network (DNN)^50^, were also implemented and evaluated. More details can be found in Supplementary Note 4.

### Implementation and evaluation of models and training strategies

#### Implementation of computational models

To fairly compare the performances of different compound activity prediction methods including both classical machine learning and deep learning algorithms, we implemented a unified training and evaluation framework for these methods. The machine learning models including RF, GBT, SVM, and DNN were implemented with Scikit-learn^70^. The deep learning models were implemented using PyTorch^71^. For those methods originally implemented using PyTorch, we adopted their source code with the necessary minimal modification to fit into our implementation framework. For those methods originally implemented using other deep learning frameworks (such as Keras^72^ or TensorFlow^73^), we implemented the PyTorch version of these methods according to their papers and source code. We adopted an Adam optimizer^74^ or a specific one originally used by these methods for model training. For computational models using the pre-trained feature representation approaches, including DeepCPI^40^, TransformerCPI^45^, and MolTrans^46^, we directly adopted the pre-trained models from the original papers.

#### Hyper-parameter selection

We optimized the learning rate and weight decay parameters for all the deep learning models using grid search, including DeepCPI, DeepDTA, DeepConvDTI, Tsubaki et al., GraphDTA, MONN, TransformerCPI, and MolTrans. More specifically, the hyper-parameter search was conducted through 5-fold cross-validation on the training assays of the VS-Kinase task. The learning rates from {0.001, 0.0005, 0.0001} and weight decay rates from {0, 0.000001, 0.00001} were considered in the calibration process. The best hyper-parameter was selected based on the average per-assay PCC for each model. The optimized hyper-parameters were used to train the models on all the VS and LO tasks in the zero-shot scenario.

In the few-shot scenario, the hyper-parameters were adjusted according to different training strategies. The same set of hyper-parameters in the zero-shot scenario was adopted for pre-training and fine-tuning, and re-training. For meta-learning, an empirical set of hyper-parameters (learning rate 0.001 and weight decay 0.000001) was applied for all three evaluated deep-learning methods. For multi-task learning, we adopted the hyper-parameters of the evaluated models from the original paper.

#### Model training in the zero-shot scenario

The computational models in the zero-shot scenario were trained on the training assays over five repeats. More specifically, the training assays were first split into five folds using the new-protein and new-assay split schemes for VS and LO tasks, respectively. Then, in each repeat, one of the five folds was used as the validation set and the remaining four folds as the training set. The resulting five models were then applied to the test assays for evaluation. The deep learning-based models were trained using the mean-square error (MSE) loss. The early-stop technique was employed to determine the training epochs and prevent over-fitting, with a maximal of 500 epochs and a patience argument of 20 epochs, using the metric of average per-assay PCC on the validation set.

#### Model training with different training strategies in the few-shot scenario

The strategies for the few-shot scenario are categorized based on how the labeled data are used for training. More specifically, in the zero-shot scenario, there is no labeled data relevant to the test assay. The training set only contains the activities of other proteins. While in the few-shot scenario, some labeled data, i.e., support set, are available for model training. After obtaining the training set and support set for a specific test assay, we can define methods or strategies in the few-shot scenario. In CARA, there are six strategies considered for the few-shot scenario, including pre-training, pre-training and fine-tuning, re-training, QSAR, meta-learning, and multi-task learning. As for the pre-training strategy, only the training set is used for training a model, while the support set is not used. For the pre-training and fine-tuning strategy, the model is first trained using the training set and then fine-tuned with the support set. In fact, the model of pre-training and fine-tuning can be obtained by fine-tuning the pre-training model with the support set. The re-training strategy combines the training set and the support set together to train the models. The only difference between re-training and pre-training is whether the support set is used in the training process. QSAR method only takes the support set for training and ignores the training set. Meta-learning has some similarities to the pre-training and fine-tuning strategy in the sense that they both have a (meta-)pre-training stage and a (meta-)fine-tuning stage, which leverages the training set and the support set, respectively. The difference between these two strategies mainly lies in the loss function. To be specific, the pre-training and fine-tuning strategy directly optimizes the objective of activity regression (e.g., mean squared error between predictions and true labels). In comparison, the meta-learning strategy aims to find the best network parameters that can optimize the objective after several steps of meta-fine-tuning. As a result, a pre-trained model may perform well and it can be further improved after fine-tuning. On the contrary, a meta-trained model may not perform well but it can be improved rapidly after several steps of meta-fine-tuning^26^. The multi-task learning strategy also makes use of both training and support sets but adopts a multi-task scheme, which divides the model parameters into shared and task-specific ones^27^.

In the few-shot scenario, each combination of models and training strategies was also trained over five repeats. More specifically, for the QSAR strategy, one model for each test assay was trained using the samples in the support set, and this process was repeated five times. For the pre-training and fine-tuning strategy, the pre-trained models adopted from the zero-shot scenario were fine-tuned using the support set individually for each test assay. For the meta-learning strategy, the models were first meta-trained using the selected training assays and then meta-fine-tuned on the support set individually for each test assay. Here, the selected training assays were those training assays with no less than 100 samples. The models trained on the selected assays turned out to achieve better performances than those using all the training assays. In each epoch of meta-training, 1000 assays were randomly sampled with replacement from the selected training assays, and at most 1000 samples were sampled for each assay, with 50 samples as the query set and the rest samples as the support set. For the multi-task learning strategy, the models were designed to have both shared and task-specific parameters and trained on the same selected training assays as in meta-learning and all the support sets of test assays. In each repeat of fine-tuning, meta-fine-tuning, and multi-task training, the support set was split into five folds, and four of them were used for training or tuning the models while the rest was used for validation. For the re-training strategy, the training data were the combination of the support sets and the same selected training assays as in meta-learning and multi-task learning, for a fair comparison between different strategies. The combined training data were then split into five folds for training and evaluating the re-training models.

### Analysis on the relation between the success rate and the number of samples in the VS task

In the VS tasks, the success assays are defined as those that have at least one hit compound in the 1% or 5% top rankings of all samples. Here, the number of hits, denoted as *H*, can be considered to follow a hypergeometric distribution, which takes *n* samples from a population of size *N* with *M* positive samples. By taking *n* samples from the population randomly without replacement, the expectation value of the number of hits is ${\mathbb{E}}(H)=n\frac{M}{N}$. In CARA, the number of hits *H* in the population and the number of token samples *n* are both defined by a threshold *p* (1% or 5%), that is, *M* = ⌈*pN*⌉, *n* = ⌈*pN*⌉, where ⌈⋅⌉ stands for the ceil operation. Therefore, we have ${\mathbb{E}}(H)=n\frac{M}{N}=\frac{{\lceil pN\rceil }^{2}}{N}\approx {p}^{2}N$. If we choose *p* = 1%, the expectation will be greater than one (i.e., success in the VS task) when *N* > 10, 000. If we choose *p* = 5%, the expectation will be greater than one when *N* > 400.

### Analyses on in-distribution and out-of-distribution data

To illustrate how the in-distribution (ID) and out-of-distribution (OOD) data can influence the performances of training strategies in the VS and LO tasks, a subset of test assays as well as the corresponding ID and OOD training data were constructed as follows. First, the test assays satisfying the specific condition were selected for evaluation. That is, for every compound in the selected test assays, we can find at least one training compound with Tanimoto similarity > 0.5 with this test compound. This process resulted in 40 and 37 selected test assays for the VS-All and LO-All tasks, respectively. Second, each compound in the training assays was marked as either ID or OOD according to whether its maximal similarity to the samples in the selected test assays was > 0.5. That is, if the similarity of a training compound with at least one test compound was greater than 0.5, it was marked as an ID training sample. If the similarities of a training compound with every test compound were < 0.5, it was marked as an OOD training sample. Third, we randomly sampled the same numbers of ID and OOD training samples for each task in the final training set, resulting in 155,598 and 35,103 ID or OOD samples for the VS-All and LO-All tasks, respectively.

### Analysis of activity cliff data

#### Definition of an activity-cliff pair

A pair of compounds (*c*_1_, *c*_2_) from the same LO assay was considered an activity-cliff (AC) pair if they were from a matched molecular pair (MMP). The MMPs were identified using the MMP algorithm implemented by RDKit, with a maximal difference of 20%. In particular, an MMP can be defined as an AC pair if

$$\Big| \left(-{\log }_{10}\,{{{{{{{\rm{Activity}}}}}}}}\,({c}_{i})\right)-\left(-{\log }_{10}\,{{{{{{{\rm{Activity}}}}}}}}({c}_{j})\,\right)\Big| > 1,$$

where Activity (⋅) stands for the activity value measured in terms of K_i_, K_d_, IC_50_, EC_50_, or Potency in nM. Otherwise, it was defined as a non-AC pair.

#### Definition of an activity-cliff compound

To describe the potential of forming AC pairs of a compound *c*_*i*_, we defined a discontinuity score to describe the activity landscape near the compound *c*_*i*_:

$${{{{{{{\rm{Disc}}}}}}}}({c}_{i})=\frac{{\sum }_{{c}_{j}\in {{{{{{{\rm{Neighbor}}}}}}}}({c}_{i})}\,{{{{{{{\rm{Sim}}}}}}}}\,({c}_{i},{c}_{j})\left| \left(-{\log }_{10}\,{{{{{{{\rm{Activity}}}}}}}}\,({c}_{i})\right)-(-{\log }_{10}{{{{{{{\rm{Activity}}}}}}}}({c}_{j}))\right|}{\Big| \,{{{{{{{\rm{Neighbor}}}}}}}}\,({c}_{i})\Big| },$$

where Neighbor(*c*_*i*_) stands for the set of compounds that can form MMPs with *c*_*i*_, and Sim(*c*_*i*_, *c*_*j*_) stands for the Tanimoto similarity between molecular fingerprints of *c*_*i*_ and *c*_*j*_. Then, a compound *c*_*i*_ was defined as an AC compound if Disc(*c*_*i*_) > 1; Otherwise it was considered a non-AC compound.

### Comparison with molecular docking-based methods

#### Selecting subsets of assays with available protein structures

We first obtained the available protein structures with known ligand binding pockets from the Protein Data Bank (PDB) database^64^. As the VS-Kinase and LO-Kinase tasks contained the largest fractions of proteins with structural data, we mapped the proteins in their test assays to the protein structures in the PDB database according to their UniProt IDs. The test assays in which the target protein can be mapped to at least one PDB structure were reserved. Then, the compounds in the matched test assay were compared against the ligands in the mapped PDB structures. For those assays with at least one compound matching a ligand in the PDB structures (i.e., the Tanimoto similarity of their molecular fingerprints was equal to one), we reversed these assays for evaluation and obtained the corresponding protein structures and ligand binding pockets for molecular docking. If multiple protein structures and pockets were matched, we selected the one with the best resolution. In the end, there were 12 out of 58 assays and 11 out of 54 assays used for molecular docking for the VS-Kinae and LO-Kinase tasks, respectively.

#### Protocols of running molecular docking-based methods

We selected several commonly used docking software including AutoDock Vina^65^, LeDock^67^, and iDock^66^ as the baseline docking methods to evaluate their performances in the VS-Kinase and LO-Kinase tasks. The data pre-processing and docking processes were conducted through following the corresponding official tutorials of these docking methods. More specifically, we first obtained the .pdb files from the PDB database^64^ and obtained the pocket centers according to the mean coordinates of their ligand atoms. For AutoDock Vina and iDock, we removed the HETATM records in the .pdb files and used the ADFR software suite^75^ to prepare the .pdbqt files for proteins. Then, the compounds were embedded from the SMILES format to 3D structures as. sdf files using RDKit^76^ and then transformed to. pdbqt files using the Meeko software (https://github.com/forlilab/Meeko). We utilized AutoDock Vina to generate the randomly initialized poses for those compound structures and then used them as the inputs for AutoDock Vina and iDock. For LeDock, we used the LePro software (http://www.lephar.com/software.htm) to prepare the .pdb files for compounds. After that, we ran the three docking methods with the same pocket centers and box sizes (20 Å). Finally, for each docking method, we adopted the energy score of the best output pose for each sample as the corresponding activity prediction result.

### Supplementary information

Supplementary Information

Description of Additional Supplementary Files

Supplementary Data 1

Supplementary Data 2

## Data Availability

The data of CARA benchmark are available in Zenodo (10.5281/zenodo.11063965). The data supporting the findings of this study are available within the article and its Supplementary Information file. The numerical source data of main figures are available in Supplementary Data 2. All other data are available from the corresponding author upon reasonable request.

## Code availability

The source code implemented in this study can be found in our GitHub repository (https://github.com/tiantz17/CARA) and Zenodo (10.5281/zenodo.11125010).
