# Supplementary material for: Benchmarking compound activity prediction for real-world drug discovery applications
Source: Commun Chem. 2024 Jun 4;7:127. doi: 10.1038/s42004-024-01204-4 (PMC11150475; doi:10.1038/s42004-024-01204-4)
Supplement: Supplementary file 1 — Supplementary Information [file 42004_2024_1204_MOESM1_ESM.pdf]

Supplementary Information for "Benchmarking Compound Activity Prediction for Real-World Drug Discovery Applications"

Tingzhong Tian[1,†], Shuya Li[1,†], Ziting Zhang[2,3], Lin Chen[4], Ziheng Zou[4],
Dan Zhao[1,*], and Jianyang Zeng[1,5,*]

[1]Institute for Interdisciplinary Information Sciences, Tsinghua University, Beijing, China.

[2]Department of Automation, Tsinghua University, Beijing, China.

[3]MOE Key Laboratory of Bioinformatics, Tsinghua University, Beijing, China.

[4]Silexon AI Technology Co., Ltd., Nanjing, Jiangsu Province, China.

[5]Present address: School of Engineering, Westlake University, Zhejiang Province, Hangzhou, China.

[†]These authors contributed equally.

[*]Corresponding authors: Dan Zhao, zhaodan2018@tsinghua.edu.cn,

and Jianyang Zeng, zengjy@westlake.edu.cn.

Supplementary Note

Supplementary Note 1. Chemical space explored by CARA

One common challenge in drug discovery is to explore the large chemical space. According to the statistics, CARA contained nearly one million compounds (926,042 unique ones), which is much larger than the existing compound activity datasets, including PDBbind (19,443 compound-protein complexes) [1], Davis (72 compounds × 442 kinases) [2] and KIBA (2,068 compounds) [3], and close to the number of compounds in the raw data of BindingDB (1.2 million) [4]. We also noticed that there are unlabeled chemical datasets containing huge numbers of compounds without known activities. Although the number of all the possible chemical compounds is extremely large, there exist many similar compounds in the chemical universe. Therefore, we speculate that it could be more reasonable to use compound similarity rather than the numbers of identical compounds to assess the relationship between CARA and the known chemical spaces. Based on this, we then checked to what extent CARA can cover the chemical space of some well-known datasets related to drug discovery. To be specific, for a query dataset containing a number of chemical compounds, we first calculated the maximum similarity of each compound against all the CARA compounds, in which the similarity is calculated as the Taminoto similarity between the Morgan fingerprints of two compounds. Then, we plotted the distribution of the maximum similarity scores of all the compounds from the query dataset (Figure 12). Here, We term the maximum similarity scores as "max-sim" scores in the remaining text. If the max-sim score is high (although not equal to 1), CARA should contain at least one sample from the chemical space nearby the corresponding compound from the query dataset. As a result, for the DrugBank [5] datasets containing more than 4,000 approved drugs, the median max-sim score of those drugs is 0.73, which means that 50% DrugBank compounds have a similarity score of at least 0.73 compared with one of the CARA compounds. For a typical high-throughput screening dataset Enamine HTS collection (labeled as Enamine-HTS) [6], which contained more than one million compounds, the median max-sim score is about 0.55. For the large-scale ZINC [7] dataset of about one billion compounds, the median max-sim score is estimated as about 0.4, which means that half of the compounds in ZINC have similarity scores of no less than 0.4 compared to any of the CARA compounds. For the compound datasets that contain specific types of compounds, e.g., the nature product database NPASS [8], the median max-sim score is still 0.49, which is still a relatively high level of similarity. Therefore, we can conclude that CARA compounds can cover a considerable fraction of chemical space related to drug discovery.

Supplementary Note 2. Pharmacological profiles of the test assays in CARA and their influence on model performances

We noticed that a specific drug discovery project often aims to find effective compounds with a certain type of pharmacological effect, and this is one of the reasons for emphasizing the usage of "assay-based" activity data in this study. The activity data from a single assay were often generated under the same experimental

condition for measuring a specific type of pharmacological profiles. According to the information of original references, we first manually checked the consistency of the pharmacological profiles of the test assays in CARA, and then classified the test assays into several major categories of common pharmacological effects, that is,

- Binding: assays characterizing binding affinities of physical interactions;
- Agonism/activation: assays characterizing the agonists or activators;
- Antagonism/inhibition: assays characterizing the antagonists or inhibitors;
- Others: uncommon assays that are not the above four types.

The numbers of test assays for each pharmacological profile category are summarized in Table 10. We also investigated whether the pharmacological profiles could affect the model performances. Here, VS-GPCR and LO-GPCR datasets were used for the analysis, as suggested by the reviewer. There were mainly "agonism", "antagonism" and "binding" categories in those GPCR datasets (Table 10). Therefore, we compared the model performances on these three types of assays, and observed that there was almost no significant difference among the three groups (Figure 13). This indicated that the types of pharmacological profiles may not have much influence on the performances of the models trained on the training assays of CARA.

We also automatically annotated the training assays of the VS-GPCR and LO-GPCR datasets through matching the key words in their assay descriptions. To be specific, an assay was labeled "antagonism" if its description contained a key word like "antagonist", "antagonistic", "inhibition", or "inhibitor". An assay was labeled "agonism" if its description contained a key word like "agonist", "agonistic", "intrinsic activity", or "effective concentration". An assay was labeled "binding" if its description contained a key word like "binding", "affinity", "displace", or "dissociation constant". Then, we re-trained one of the best methods, i.e., DeepConvDTI, with the highest EF@1% or PCC score in our original tests on the VS-GPCR or LO-GPCR datasets, on three different groups of training assays measuring binding, antagonism and agonism activities, respectively. Through the evaluation on the three types of test assays, we observed that the models trained on different assays did not significantly affect the performances on the three groups of test assays (Figure 14). This result further indicated that the types of pharmacological profiles may not act as a major influential factor on the model performances.

Supplementary Note 3. Analyzing the prediction performances on the unexplored chemical space

To provide a direct understanding of how many unexplored compounds were there in the test set of our CARA benchmark, we computed the similarities of chemical compounds in the test sets compared to those in the training set. A test compound was marked "unexplored" if its maximal similarity to the training compounds was less than or equal to 0.5. Otherwise, the test compound was marked "explored". The similarity between two compounds was defined as the Tanimoto similarity of their Morgan fingerprints.

After examination, we observed that there were 60 and 63 test assays containing at least one unexplored test compound in the VS-All and LO-All tasks, respectively. As shown in Table 11, 5 and 17 test assays were found to have over 50% of unexplored test compounds in the VS-All and LO-All tasks, respectively. These test assays can be used to mimic the situation where novel compounds are to be discovered. We then split the test assays into two groups based on the fraction of unexplored test compounds with a threshold of 50%, i.e., one group of test assays with the fraction of unexplored compounds $<$ 50%, and the other group of test assays with the fraction of unexplored compounds $\geq$ 50%.

As shown in Figure 15, the pre-training strategy on the group with more unexplored compounds (i.e., unexplored compounds $\geq$ 50%) achieved a lower EF@1% score on the VS-All task, compared with the group with less unexplored compounds (unexplored compounds $<$ 50%). However, the difference between the two scores was not significant according to the Student's t test. Similar results were also observed for the other two strategies, i.e., pre-training and fine-tuning as well as meta-learning. We speculated that this was probably because there were only a few assays in the group with more unexplored compounds (i.e., unexplored compounds $\geq$ 50%).

As for the LO-All task, we observed that the pre-training strategy on the group with more unexplored compounds (i.e., unexplored compounds $\geq$ 50%) achieved a significantly lower PCC compared with the group with less unexplored compounds (i.e., unexplored compounds $<$ 50%). This result suggested that the more unexplored compounds in the test set may lead to higher difficulty of the task. We also observed that the QSAR strategy achieved better PCC scores on both groups compared with the pre-training strategy, which was consistent with the conclusions drawn in Section 2.4. In addition, there was no significant difference in the PCC scores achieved by the QSAR strategy on these two groups, suggesting that the QSAR strategy was not sensitive to the existence of unexplored compounds. Taken together, the above observation indicated that the QSAR strategy can improve the performances of compound activity prediction models even on unexplored compounds.

Supplementary Note 4. Baseline methods for compound activity prediction

To evaluate the compound activity prediction power under our CARA benchmark, we selected several representative compound activity prediction methods as the baselines. Generally, the compound activity prediction methods usually predict the activity label of a chemical compound against a protein target. The activity prediction task can be either a classification task where the activity labels are binary (active or inactive), or a regression task where the activity labels are real values. In this work, we mainly focused on the regression task.

Based on the input information, the compound activity prediction methods can be categorized into two classes, i.e., the "two-tower" style or the quantitative structure-activity relationship (QSAR) style. A method in the two-tower style takes both compound and protein features as inputs and predicts the activity based on the information from both sides. On the contrary, the method in the QSAR style only takes the features of a

compound as input and predicts its activity using only compound information. Obviously, the methods in the two-tower style can be trained on the datasets containing multiple proteins, while those methods in the QSAR style can only be trained on the dataset of a single protein.

Here, we considered the compound activity prediction methods in both styles. For methods in the two-tower style, we selected DeepCPI [9], DeepDTA [10], DeepConvDTI [11], GraphDTA [12], MONN [13], Tsubaki et al. [14], MolTrans [15], and TransformerCPI [16]. For methods in the QSAR style, we selected random forest (RF) [17], gradient boosting tree (GBT) [18], support vector machine (SVM) [19], and deep neural network (DNN) [20]. Below we summarize the description of each selected compound activity prediction method.

DeepCPI utilizes a two-stage strategy for predicting compound activities. Compound and protein features are first extracted and compressed using singular value decomposition (SVD) based on the Morgan fingerprints of compounds and a 3-mer feature encoding scheme of proteins. Then, in the second stage, fully-connected layers taking the concatenated features of both compounds and proteins as inputs are trained to predict their activities.

DeepDTA predicts the compound activities in an end-to-end manner. The features of input molecules are learned through convolutional neural networks (CNNs) from the SMILES (simplified molecular-input line-entry system) strings of compounds and the primary sequences of proteins. Then, fully-connected layers are used to predict the activities.

DeepConvDTI also predicts the compound activities in an end-to-end manner. The protein features are learned with a CNN-based architecture. The compound features are extracted from Morgan fingerprints, followed by fully-connected layers. Then, the protein and compound features are concatenated and fed into additional fully-connected layers to predict the activities.

GraphDTA improves DeepDTA through utilizing graph neural networks (GNNs) to encode the molecular graphs of compounds, in which atoms and bonds of a compound are considered as nodes and edges in a graph, respectively. The remaining parts, including protein feature extraction and activity prediction, are the same as in DeepDTA.

MONN predicts the compound activities with an additional objective of predicting the non-covalent interactions between protein residues and compound atoms. A graph warp module is introduced by MONN to encode the compound features. The protein features are extracted by a CNN module. Then, the activities are predicted by a dual memory attention module considering the interactive patterns between compounds and proteins.

Tsubaki et al. introduced another compound protein binding affinity prediction approach. The compound features are learned using GNNs while the protein features are extracted through CNNs. Then, these features are concatenated to predict their binding affinities.

MolTrans introduces an augmented transformer encoder to learn the sub-structural patterns of compound activities. The SMILES strings and the protein sequences are first processed into sub-sequences. Then, the features of these sub-sequences are updated using a transformer encoder. After that, the interaction features between compound and protein sub-structures are extracted and used for activity prediction.

TransformerCPI employs a transformer decoder architecture to predict compound activities from the input

compound SMILES strings and protein sequences. More specifically, the amino acid sequences of proteins are used as the input to the transformer encoder. Then, the SMILES sequences of compounds and the protein features learned by the transformer encoder are fed into the transformer decoder to generate the interaction features. Then the interaction probabilities or activity values can be predicted using fully-connected layers.

The **QSAR models** used in this work, including RF, SVM, GBT, and DNN, all predict the compound activities using the Morgan fingerprints as inputs. The protein information does not serve as model input. During training, the compounds in the same assay, i.e., with the same protein target, were used to train the QSAR models, and this procedure was repeated for each assay.

Supplementary Figures

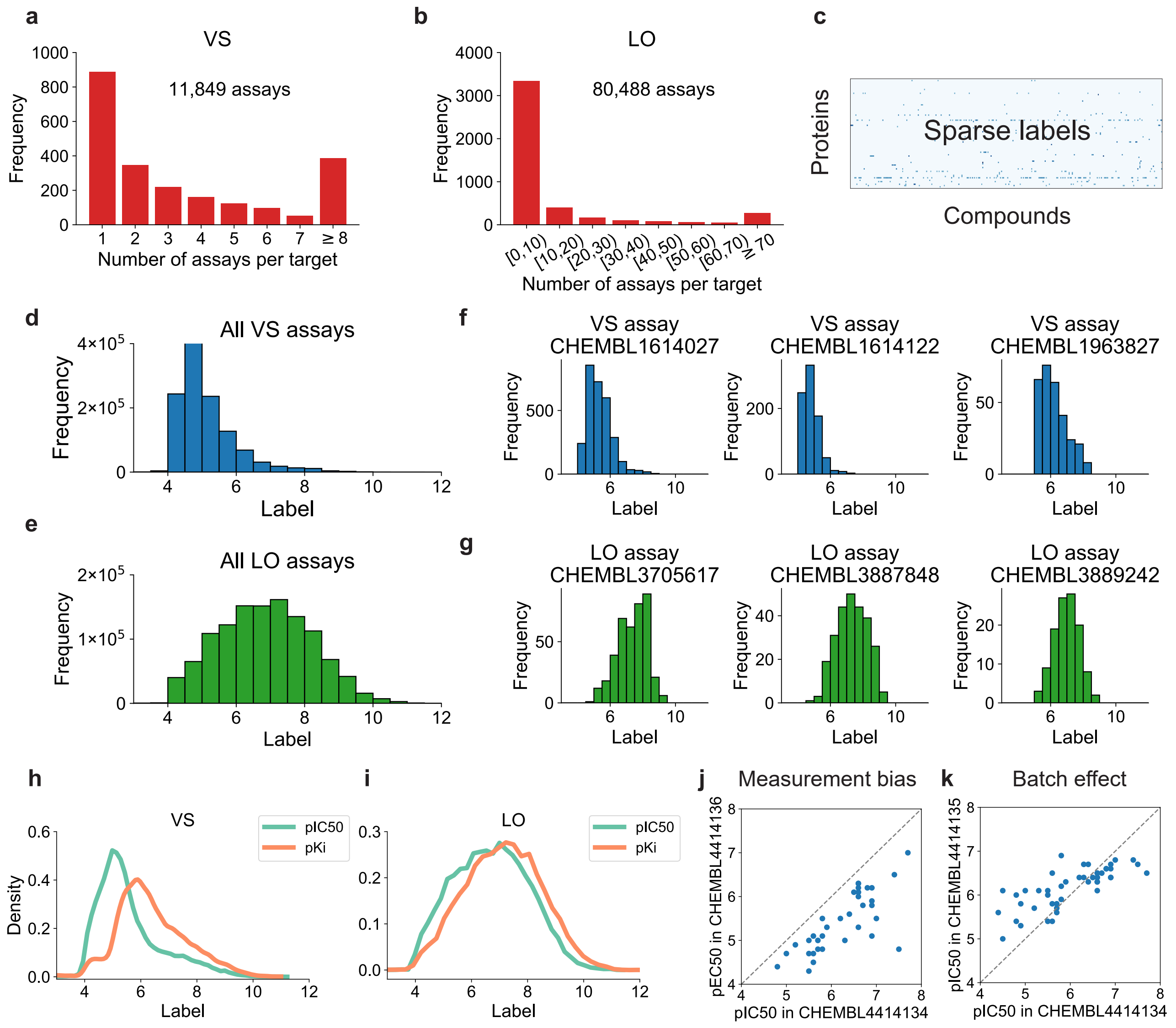

Supplementary Figure 1: Supplementary analyses on the characteristics of real-world compound activity data. **a**–**b**. Distributions of the numbers of VS (**a**) and LO (**b**) assays per target in ChEMBL. **c**. Visualization of a part of the label matrix, in which dark blue points stand for the observed activity data for the corresponding compound-protein pairs. **d**–**e**. Distributions of activity labels in all the VS (**d**) or LO (**e**) assays in ChEMBL. **f**–**g**. Distributions of activity labels in the selected VS (**f**) and LO (**g**) assays. **h**–**i**. Distributions of all the activity values (labels) that were measured by IC_{50} and K_i on the same set of compound-protein pairs for VS (**h**) and LO (**i**) assays. **j**. An example of measurement bias on the same set of compound-protein pairs from two assays measured by IC_{50} and EC_{50}, respectively. **k**. An example of batch effect on the same set of compound-protein pairs from two assays both measured by IC_{50} values.

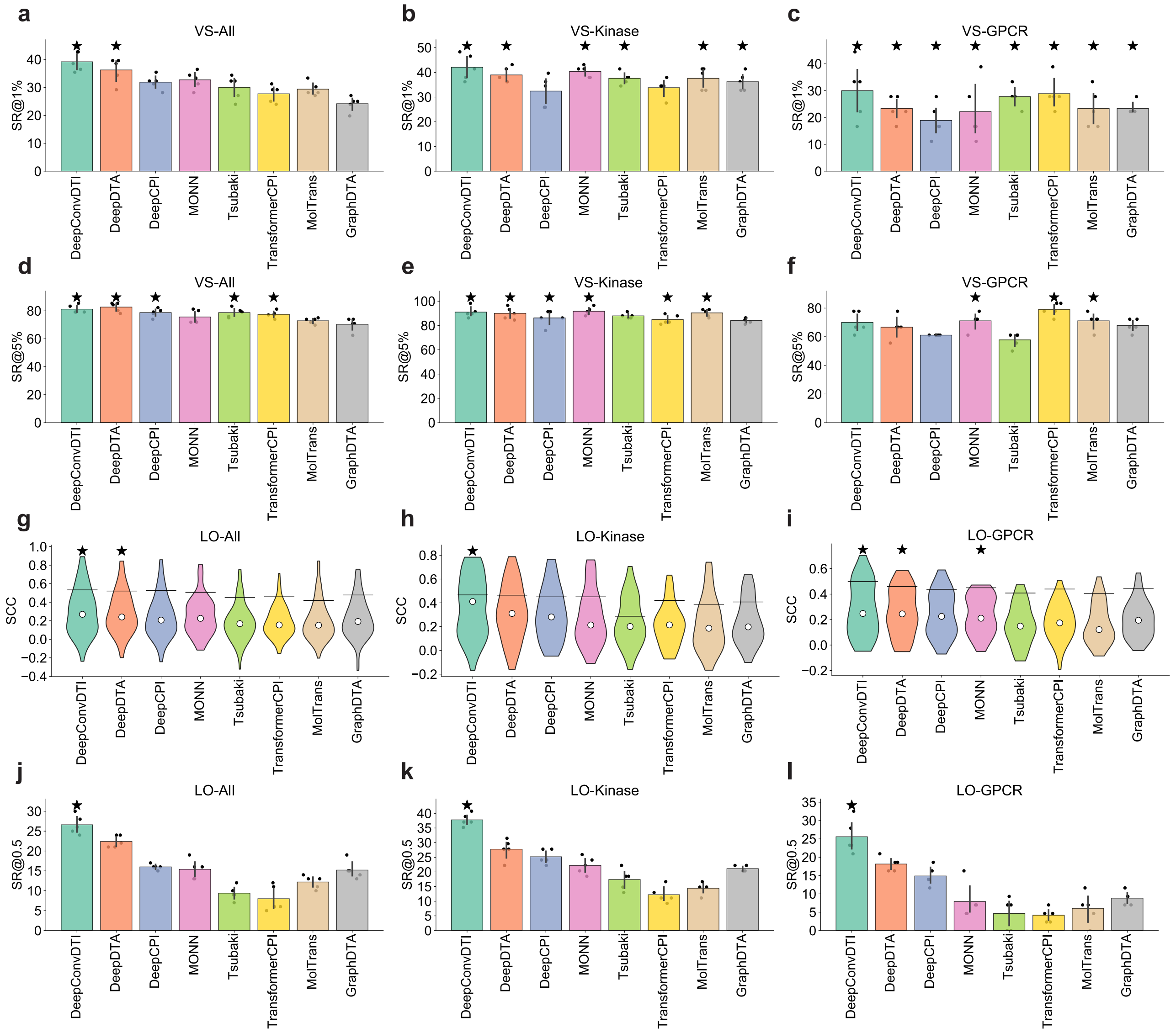

Supplementary Figure 2: Performance evaluation of different compound activity prediction models with success rates at top 1% (SRs@1%), per-assay enrichment factors at top 5% (EFs@5%), success rates at top 5% (SRs@5%), per-assay Spearman's correlation coefficients (SCCs), success rates with PCCs $>$ 0.5 (SRs@0.5) under the zero-shot scenario. **a**–**c**. Success rates at top 1% (SRs@1%) on the VS-All (**a**), VS-Kinase (**b**), and VS-GPCR (**c**) tasks, respectively. **d**–**f**. Success rates at top 5% (SRs@5%) on the VS-All (**d**), VS-Kinase (**e**), and VS-GPCR (**f**) tasks, respectively. **g**–**i**. Violin plots of per-assay Spearman's correlation coefficients (SCCs) on the test assays of the LO-All (**g**), LO-Kinase (**h**), and LO-GPCR (**i**) tasks, respectively. **j**–**l**. Success rates with PCCs $>$ 0.5 (SRs@0.5) on the LO-All (**j**), LO-Kinase (**k**), and LO-GPCR (**l**) tasks, respectively. The best models and those with no significant difference compared to the best ones are marked with stars. The significance levels were calculated using two-sided *t*-tests adjusted by a false discovery rate of 0.05. The error bars in **a**–**f** and **j**–**l** stand for the standard deviations over five repeats.

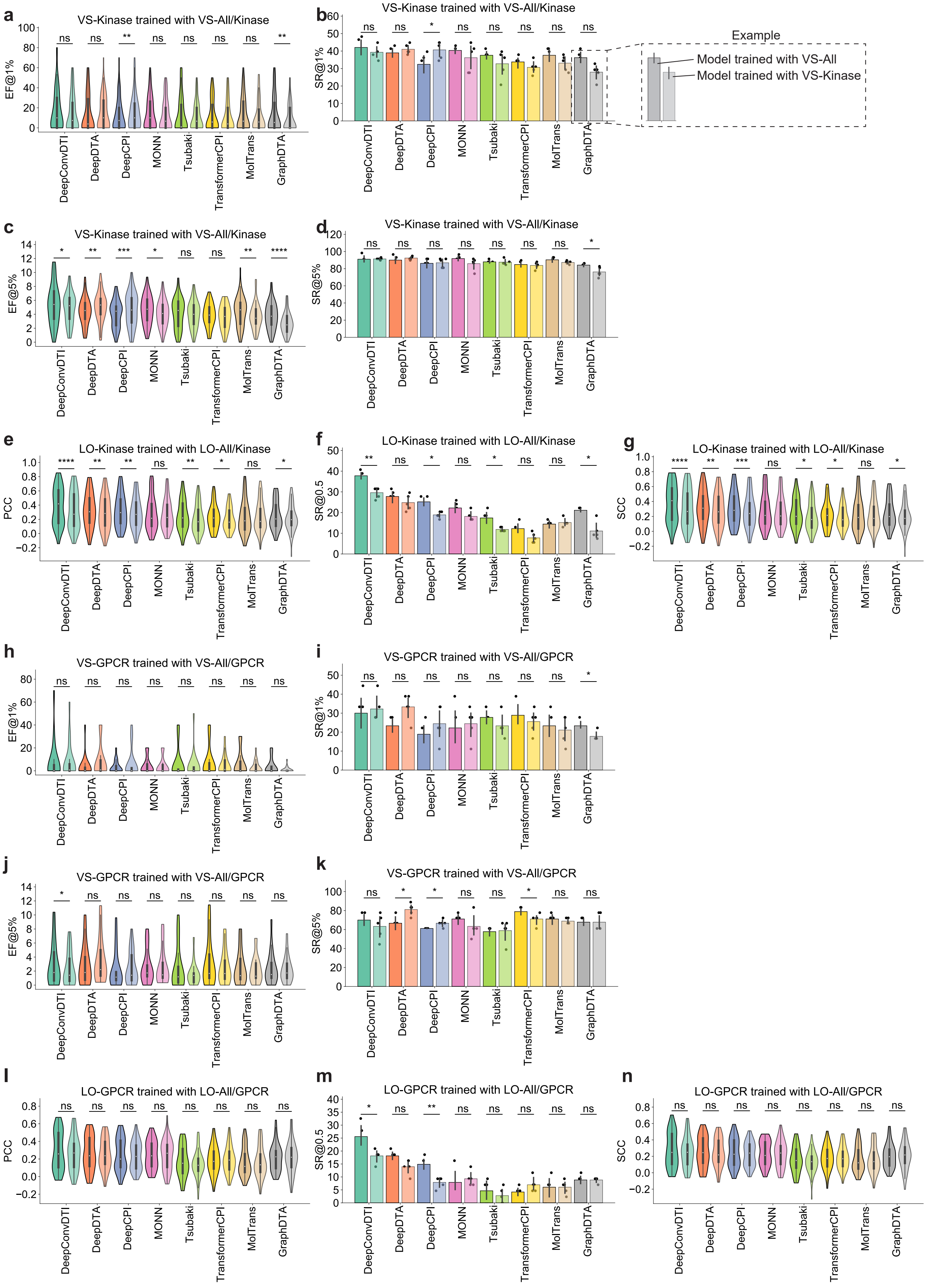

a
VS-Kinase trained with VS-All/Kinase
EF@1%
ns ns ** ns ns ns ns **
DeepConvDTI DeepDTA DeepCPI MONN Tsubaki TransformerCPI MolTrans GraphDTA
b
VS-Kinase trained with VS-All/Kinase
SR@1%
ns ns * ns ns ns ns ns
Example
Model trained with VS-All
Model trained with VS-Kinase
c
VS-Kinase trained with VS-All/Kinase
EF@5%
* ** *** * ns ns ** ****
d
VS-Kinase trained with VS-All/Kinase
SR@5%
ns ns ns ns ns ns ns *
e
LO-Kinase trained with LO-All/Kinase
PCC
**** ** ** ns ** * ns *
f
LO-Kinase trained with LO-All/Kinase
SR@0.5
** ns * ns * ns ns *
g
LO-Kinase trained with LO-All/Kinase
SCC
**** ** *** ns * * ns *
h
VS-GPCR trained with VS-All/GPCR
EF@1%
ns ns ns ns ns ns ns ns
i
VS-GPCR trained with VS-All/GPCR
SR@1%
ns ns ns ns ns ns ns *
j
VS-GPCR trained with VS-All/GPCR
EF@5%
* ns ns ns ns ns ns ns
k
VS-GPCR trained with VS-All/GPCR
SR@5%
ns * * ns ns * ns ns
l
LO-GPCR trained with LO-All/GPCR
PCC
ns ns ns ns ns ns ns ns
m
LO-GPCR trained with LO-All/GPCR
SR@0.5
* ns ** ns ns ns ns ns
n
LO-GPCR trained with LO-All/GPCR
SCC
ns ns ns ns ns ns ns ns

Supplementary Figure 3: Performance evaluation of different compound activity prediction models under the zero-shot scenario trained with the training assays from VS-All/LO-All tasks (in dark colors) or VS-Kinase/VS-GPCR/LO-Kinase/LO-GPCR tasks (in light colors). **a**. Violin plots of per-assay enrichment factors at top 1% (EFs@1%) on the test assays of the VS-Kinase task. **b**. Success rates at top 1% (SRs@1%) on the test assays of the VS-Kinase task. **c**. Violin plots of per-assay enrichment factors at top 5% (EFs@5%) on the test assays of the VS-Kinase task. **d**. Success rates at top 5% (SRs@5%) on the test assays of the VS-Kinase task. **e**. Violin plots of per-assay Pearson's correlation coefficients (PCCs) on the test assays of the LO-Kinase task. **f**. Success rates with PCC $>$ 0.5 (SRs@0.5) on the test assays of the LO-Kinase task. **g**. Violin plots of per-assay Spearman's correlation coefficients (SCCs) on the test assays of the LO-Kinase task. **h**–**n**. The same metrics as in (**a**–**g**) for VS-GPCR (**h**–**k**) and LO-GPCR (**l**–**n**) tasks, respectively. The significance levels were calculated using two-sided *t*-tests for the same models trained with different sets of assays. ****: p-value $<$ 0.0001, ***: p-value $<$ 0.001, **: p-value $<$ 0.01, *: p-value $<$ 0.05, ns: not significant. The error bars in **b**, **d**, **f**, **g**, **i**, **k**, and **m** stand for the standard deviations over five repeats.

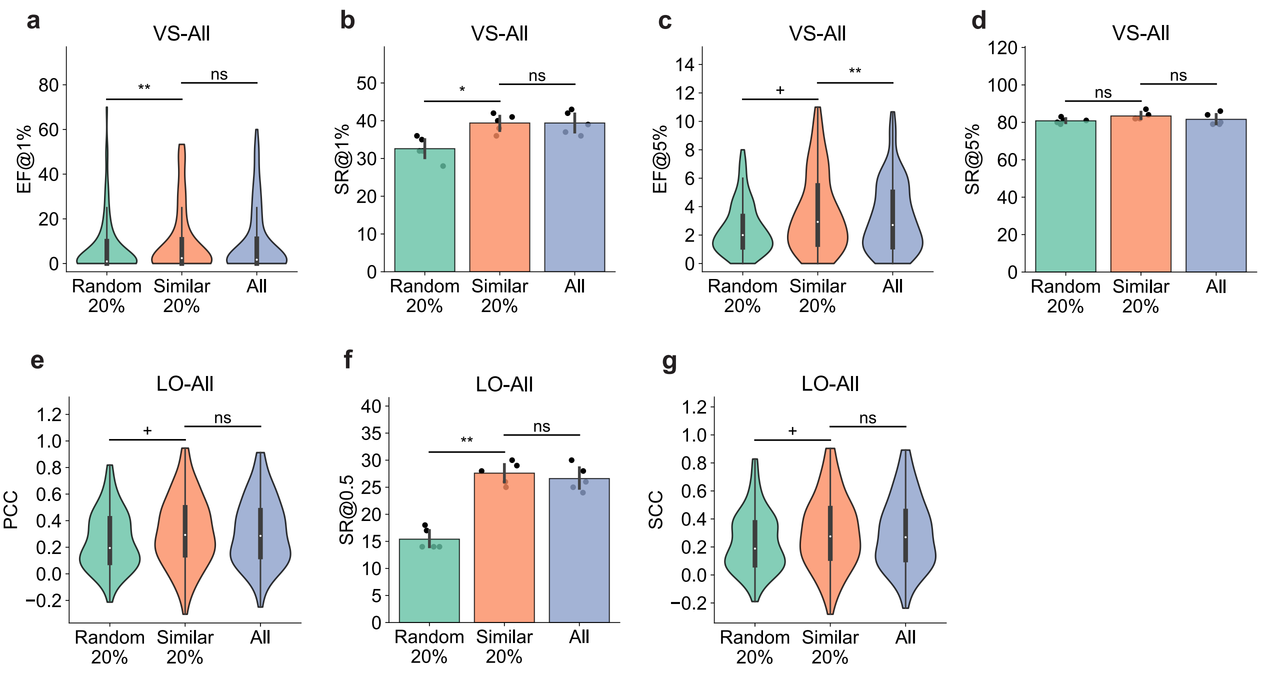

Supplementary Figure 4: Performance evaluation of DeepConvDTI under the zero-shot scenario trained with 20% of the original training samples. The training samples were either selected randomly (denoted as random) or obtained from a subset of samples that were most similar to the compounds in the test assay (denoted as similar). The models were trained separately for each test assay, i.e., one model for each test assay. **a**. Violin plots of per-assay enrichment factors at top 1% (EFs@1%) on the test assays of the VS-All task. **b**. Success rates at top 1% (SRs@1%) on the test assays of the VS-All task. **c**. Violin plots of per-assay enrichment factors at top 5% (EFs@5%) on the test assays of the VS-All task. **d**. Success rates at top 5% (SR@5%) on the test assays of the VS-All task. **e**. Violin plots of per-assay Pearson's correlation coefficients (PCCs) on the test assays of the LO-All task. **f**. Success rates with PCC > 0.5 (SR@0.5) on the test assays of the LO-All task. **g**. Violin plots of per-assay Spearman's correlation coefficients (SCCs) on the test assays of the LO-All task. The significance levels were calculated using two-sided *t*-tests for the same models trained with different sets of assays. +: p-value < 0.00001, **: p-value < 0.01, *: p-value < 0.05, ns: not significant. The error bars in **b**, **d**, and **f** stand for the standard deviations over five repeats.

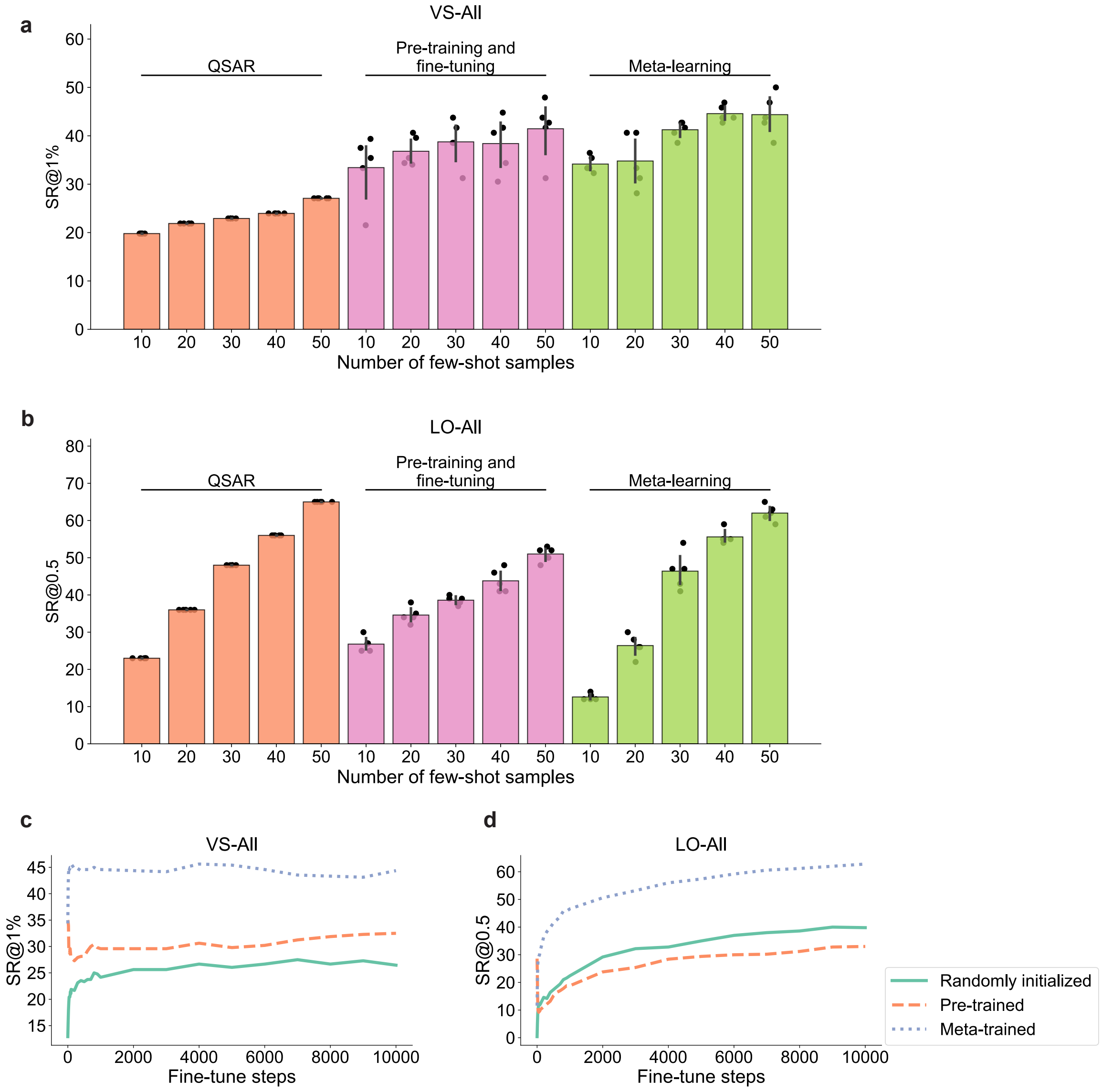

Supplementary Figure 5: Influences of the numbers of few-shot samples and fine-tuning steps on the prediction performances under the few-shot scenario. **a**. Success rates at 1% (SRs@1%) with respect to the number of few-shot samples in the VS-All task. **b**. Success rates with PCC $>$ 0.5 (SRs@0.5) with respect to the number of few-shot samples in the LO-All task. **c**. Success rates at 1% (SRs@1%) with respect to the number of fine-tuning steps in the VS-All task. **d**. Success rates with PCC $>$ 0.5 (SRs@0.5) with respect to the number of fine-tuning steps in the LO-All task. Three fine-tuning schemes using different initial models are shown, including a randomly initialized model, a pre-trained model, and a meta-trained model.

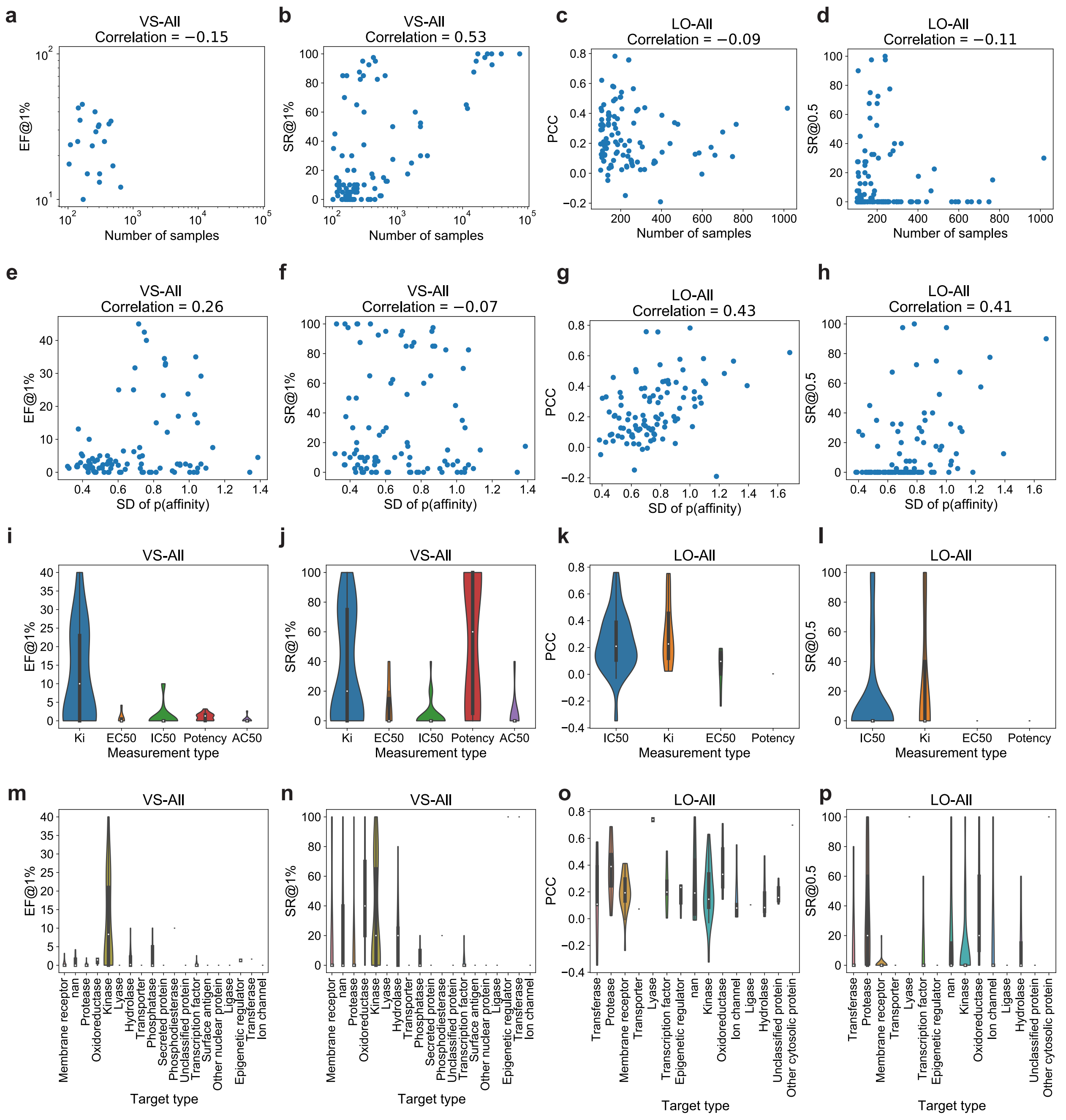

Supplementary Figure 6: The relationships between model performances measured in terms of enrichment factors at 1% (EFs@1%), success rates at 1% (SRs@1%), Pearson's correlation coefficients (PCCs), and success rates with PCC $>$ 0.5 (SRs@0.5) with the number of samples (**a**–**d**), the standard deviation (SD) of activity labels (**e**–**h**), the measurement type (**i**–**l**), and the target type (**m**–**p**) of individual assays in the VS-All and LO-All tasks, respectively.

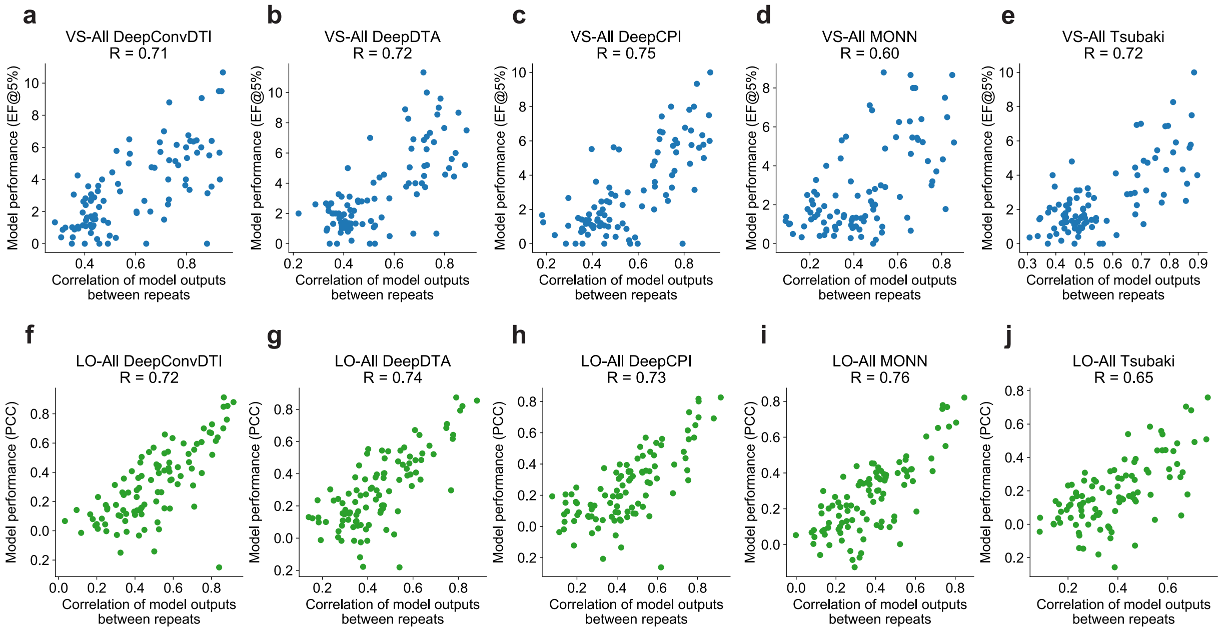

Supplementary Figure 7: The correlations of model outputs between repeats of the same method were correlated with model performances in both the VS-All (**a**–**e**) and LO-All (**f**–**j**) tasks.

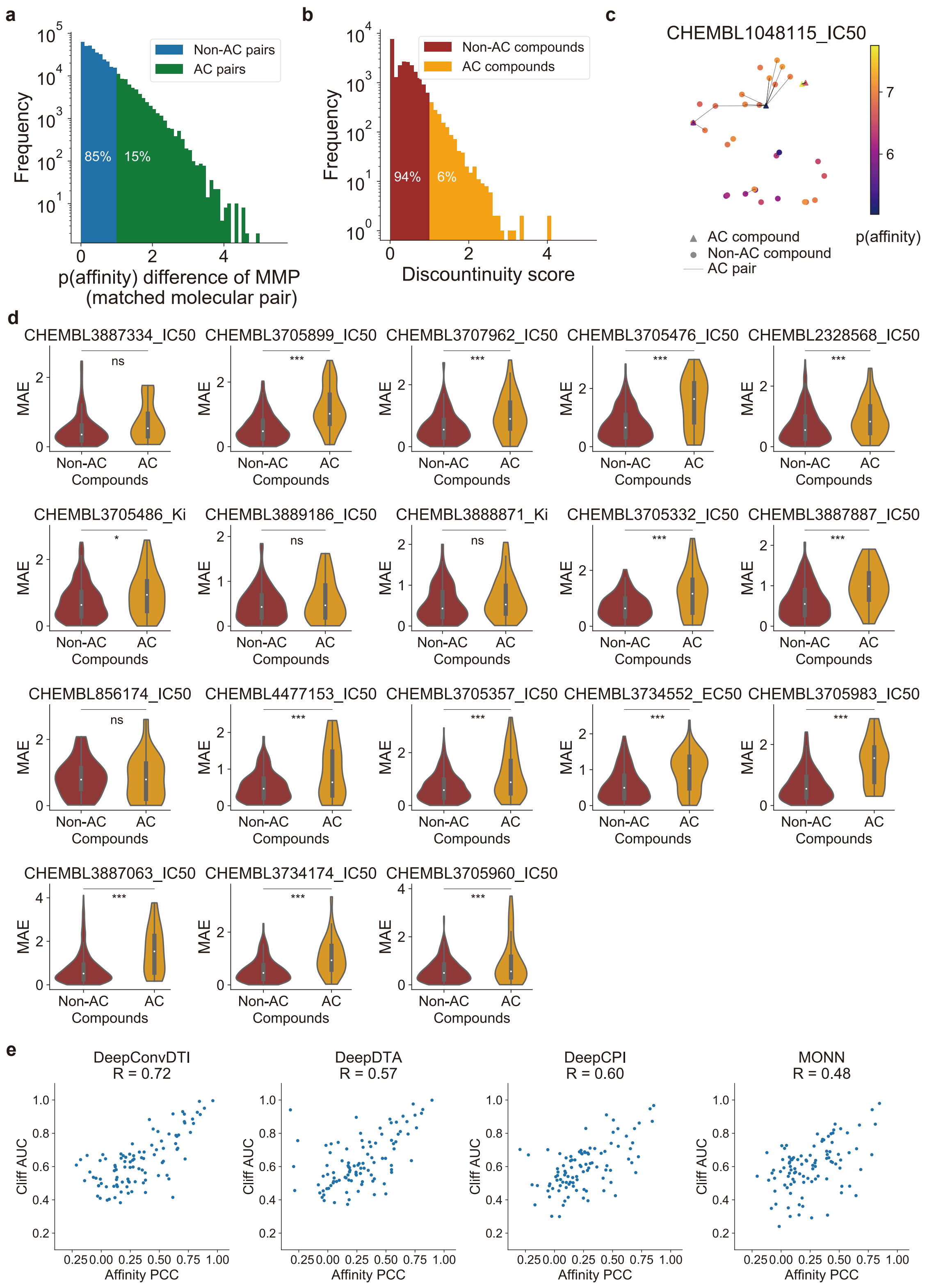
a
Frequency
Non-AC pairs
AC pairs
85%
15%
p(affinity) difference of MMP
(matched molecular pair)
b
Non-AC compounds
AC compounds
94%
6%
Discountinuity score
c
CHEMBL1048115_IC50
AC compound
Non-AC compound
AC pair
p(affinity)
d
CHEMBL3887334_IC50
CHEMBL3705899_IC50
CHEMBL3707962_IC50
CHEMBL3705476_IC50
CHEMBL2328568_IC50
CHEMBL3705486_Ki
CHEMBL3889186_IC50
CHEMBL3888871_Ki
CHEMBL3705332_IC50
CHEMBL3887887_IC50
CHEMBL856174_IC50
CHEMBL4477153_IC50
CHEMBL3705357_IC50
CHEMBL3734552_EC50
CHEMBL3705983_IC50
CHEMBL3887063_IC50
CHEMBL3734174_IC50
CHEMBL3705960_IC50
MAE
Non-AC
AC
Compounds
e
DeepConvDTI
R = 0.72
DeepDTA
R = 0.57
DeepCPI
R = 0.60
MONN
R = 0.48
Cliff AUC
Affinity PCC

Supplementary Figure 8: Performance analyses of different models on activity cliff data. **a**. The frequencies of activity cliff (AC) and non-AC pairs in the test assays of the LO-All task. The frequencies were computed according to the difference of p(activity) in the matched molecular pairs (MMPs). Refer to the main text and Methods for more details about the definition of MMPs. **b**. The frequencies (i.e., discontinuity scores) of AC and non-AC compounds in the test assays of the LO-All task. See Methods for more details about the definition of AC compounds. **c**. Visualization of a subset of compounds in an example assay containing AC pairs and AC compounds. **d**. Performances of the best model under the few-shot scenario (i.e., DeepConvDTI-c with multi-task learning) on AC and non-AC compounds evaluated in terms of the mean absolute error (MAE). All the test assays with more than 20 AC compounds from the LO-All task are shown. The significance levels were calculated using two-sided *t*-tests adjusted by a false discovery rate of 0.05. ***: p-value < 0.001, **: p-value < 0.01, *: p-value < 0.05, ns: not significant. **e**. Scatter plots of Pearson's correlation coefficients (PCCs) from the affinity prediction task and area under the receiver operator characteristic curve (AUROC) scores from the cliff prediction task in the test assays of the LO-All task.

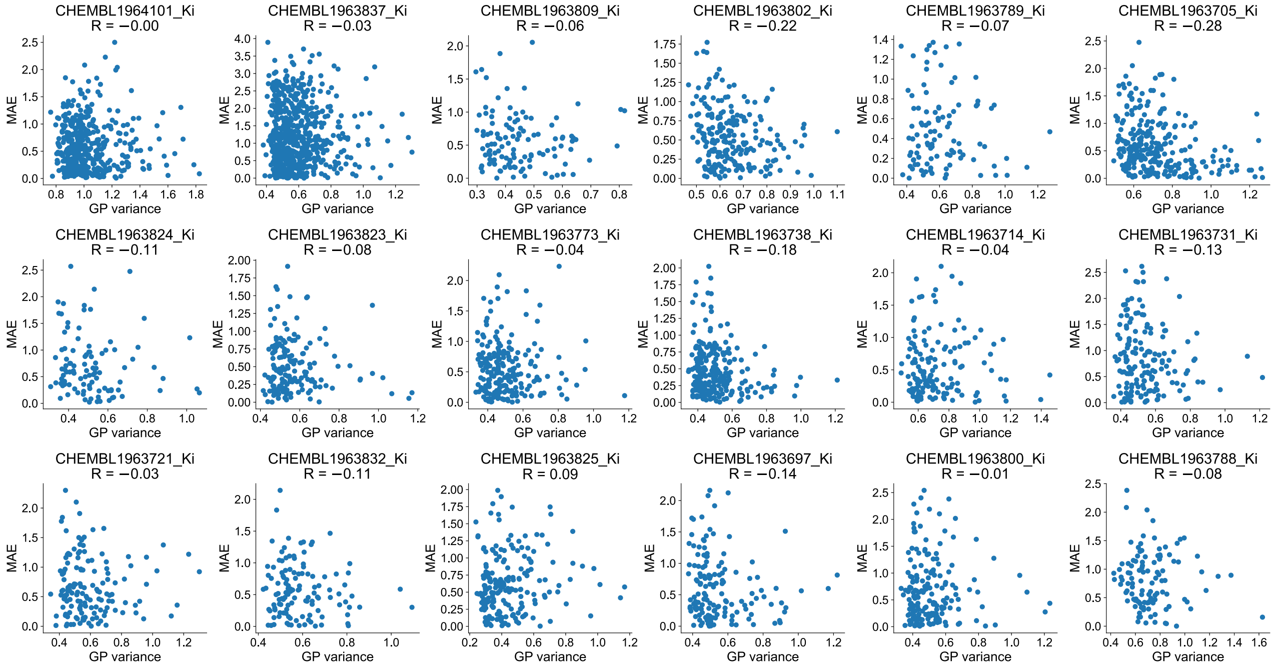

Supplementary Figure 9: The relationship between the variance of the Gaussian process (GP) model and the mean-absolute-errors (MAEs) of samples on several example assays in the VS-Kinase task.

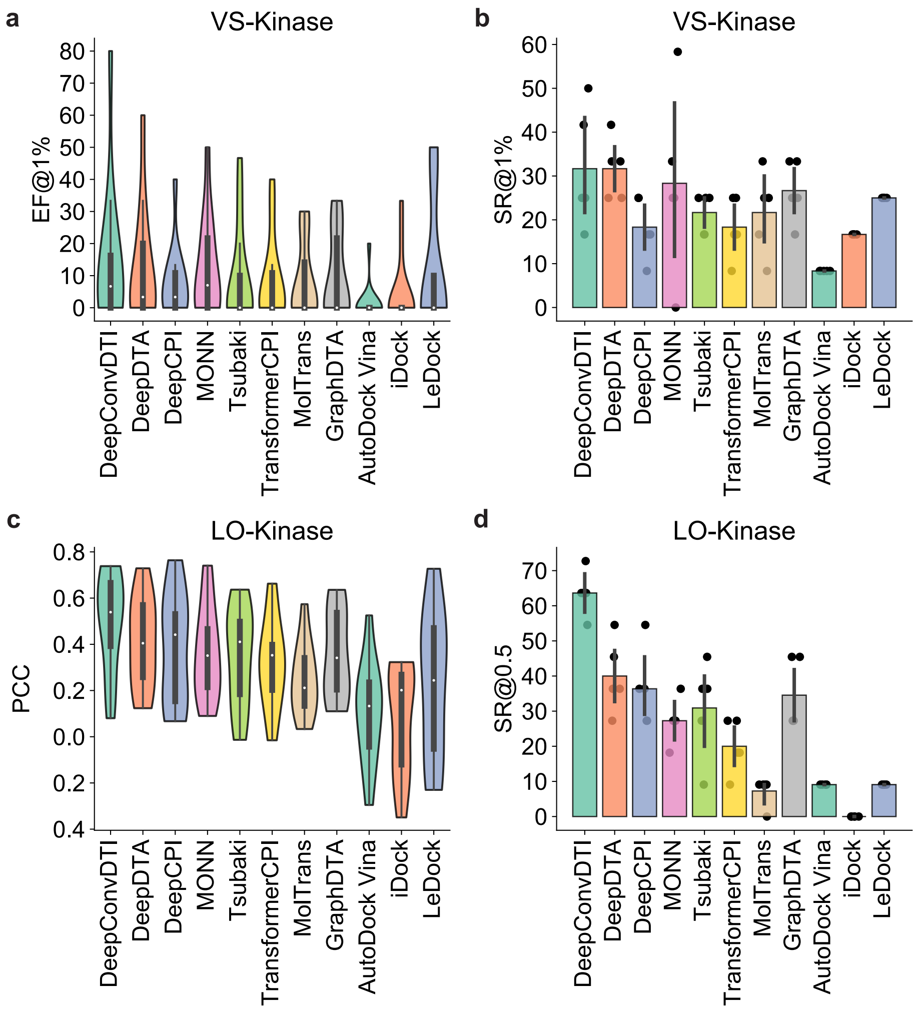

Supplementary Figure 10: Performance comparisons of the deep learning-based and the molecular docking-based methods on the subsets of test assays with known protein structures in the VS-Kinase and LO-Kinase tasks. **a**–**b**. Violin plots of per-assay enrichment factors at 1% (EFs@1%, **a**) and success rates at 1% (SRs@1%, **b**) of the deep learning-based models trained under the zero-shot scenario and the molecular docking-based methods for the VS-Kinase task. **c**–**d**. Violin plots of per-assay Pearson's correlation coefficients (PCCs, **c**) and success rates with PCC $>$ 0.5 (SRs@0.5, **d**) of the deep learning-based models trained under the zero-shot scenario and the molecular docking-based methods for the LO-Kinase task. The error bars in **b** and **d** stand for the standard deviations over five repeats.

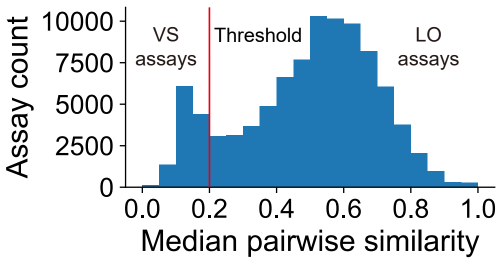

Supplementary Figure 11: Histogram of the median pairwise compound similarities for each assay. The pairwise similarities between the compounds in individual assays were first calculated, and then the median value of the pairwise similarities was obtained for each assay. A threshold of 0.2 was used to distinguish two types of assays, i.e., VS and LO.

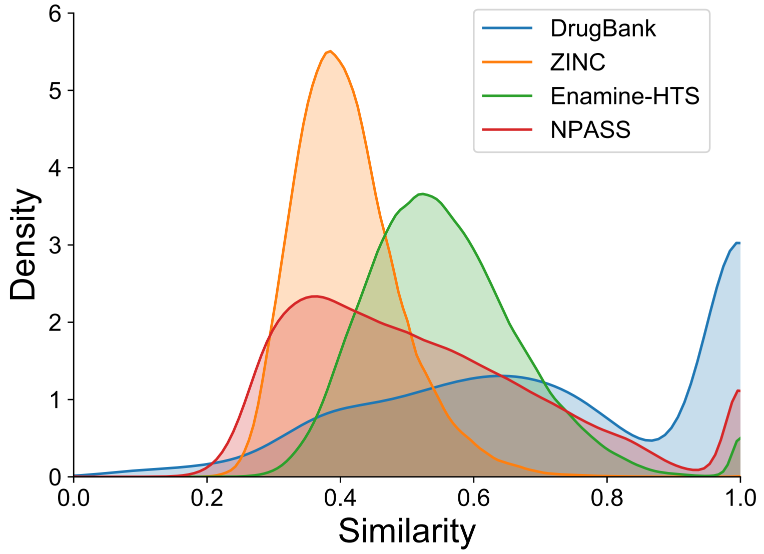

Supplementary Figure 12: Distributions of maximum similarity scores of compounds from the four drug-related datasets, which were calculated and maximized over all the CARA compounds. The distributions of DrugBank and NPASS were calculated on their 4,103 and 96,236 compounds, respectively. The distributions of the large-scale ZINC and Enamine-HTS datasets were estimated through randomly sampling 100,000 compounds from each dataset.

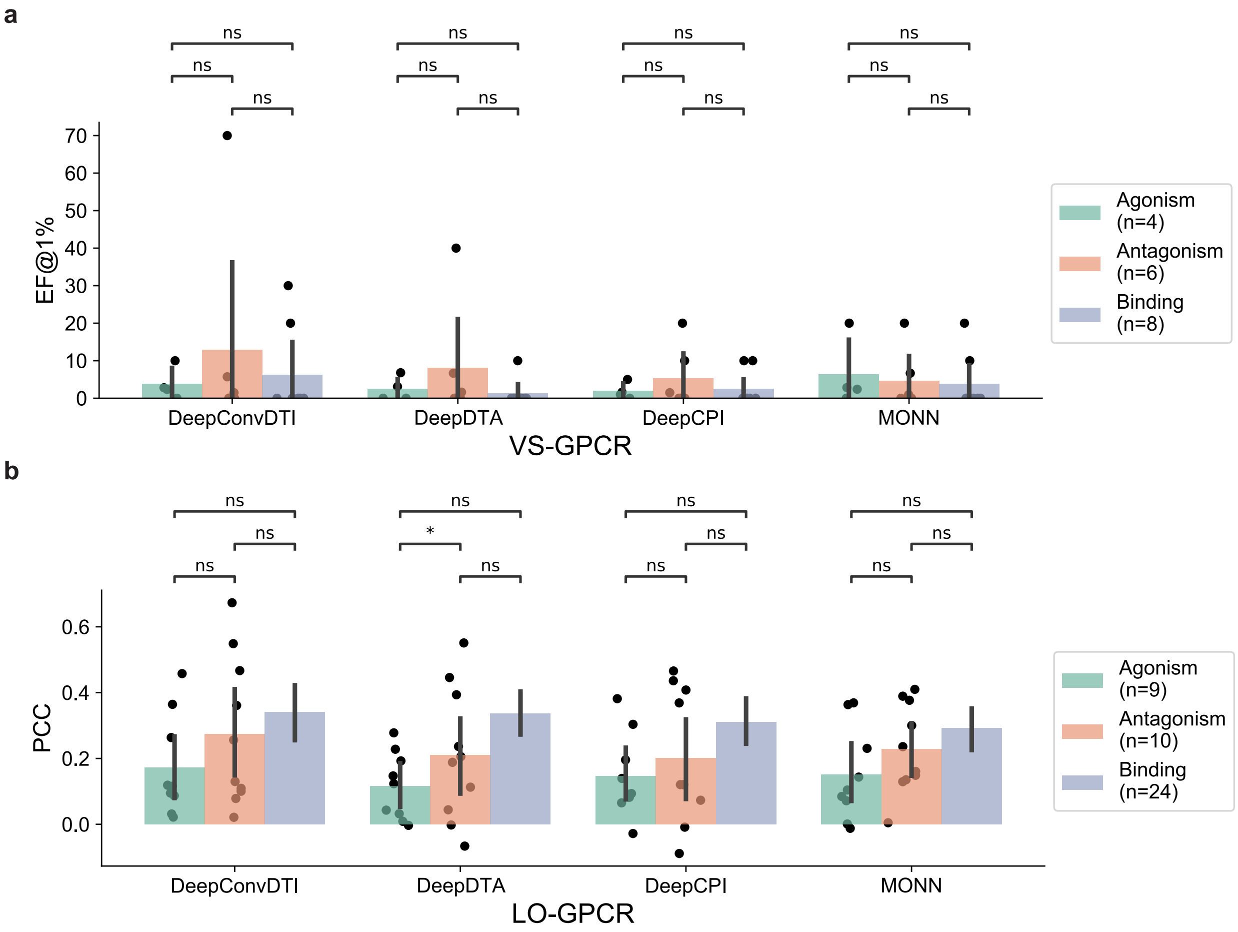

Supplementary Figure 13: Performances of different compound activity prediction methods evaluated on the VS-GPCR (**a**) and LO-GPCR (**b**) tasks, repectively, under the zero-shot scenario. The test assays were grouped according to the measured pharmacological profiles. ns: not significant, calculated using Student's t tests.

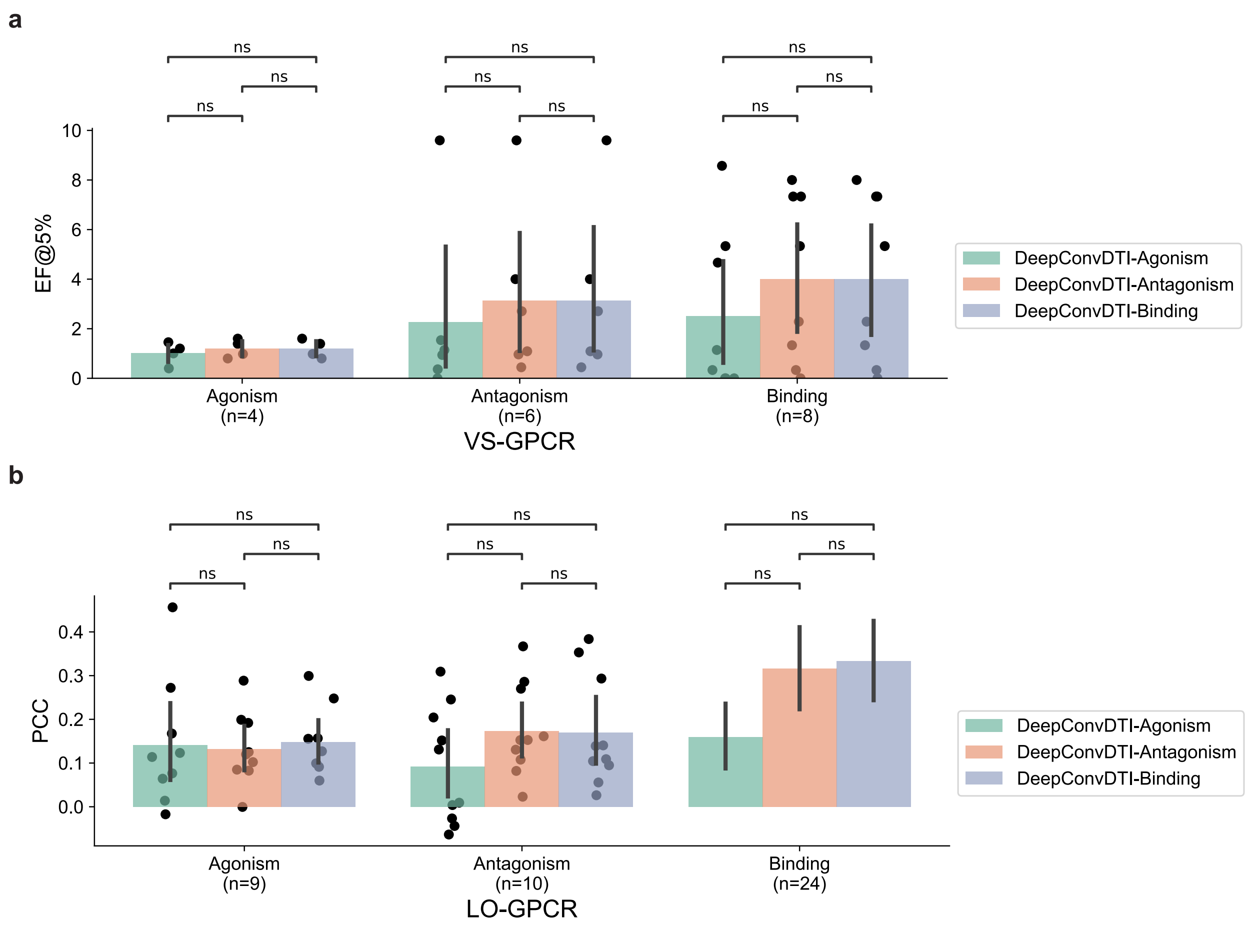

Supplementary Figure 14: Performance of the compound activity prediction method DeepConvDTI trained and evaluated using assays from different pharmacological profiles on the VS-GPCR (**a**) and LO-GPCR (**b**) tasks, respectively, under the zero-shot scenario. Both training and test assays were grouped according to the measured pharmacological profiles. ns: not significant, calculated using Student's t tests.

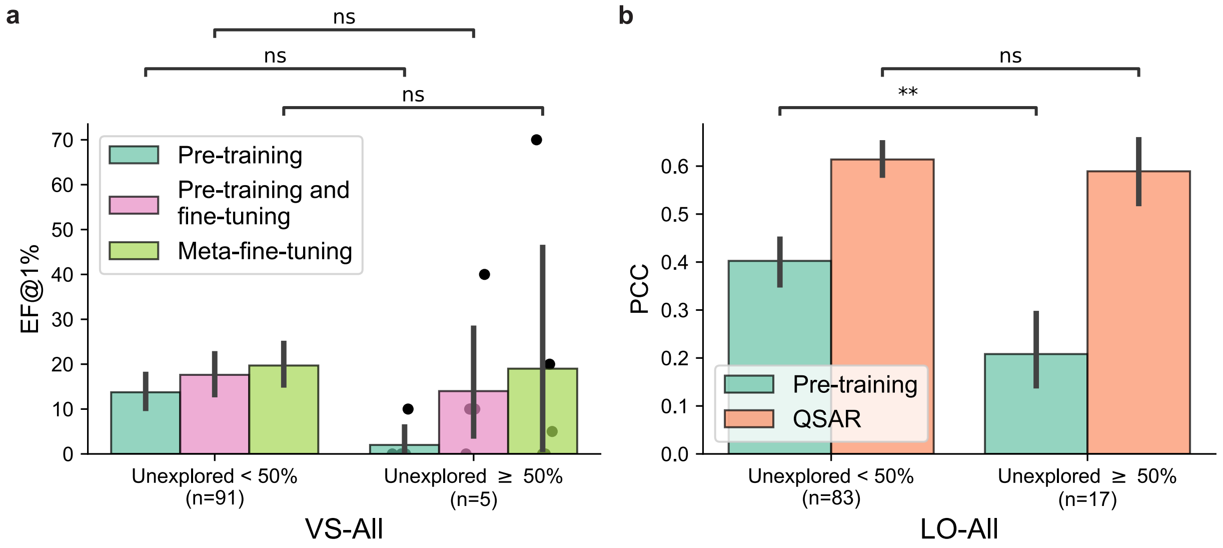

Supplementary Figure 15: Averaged performances of different training strategies grouped according to the fractions of unexplored compounds on the VS-All (**a**) and LO-All (**b**) tasks, respectively.

Supplementary Tables

Supplementary Table 1: Example of an assay with 10 samples.

Assay ID	Target ID	Measurement Type	Compound ID	Activity
CHEMBL3241595	CHEMBL203	IC50	CHEMBL1229592	8.36
CHEMBL3241595	CHEMBL203	IC50	CHEMBL3237929	6.20
CHEMBL3241595	CHEMBL203	IC50	CHEMBL3237930	6.06
CHEMBL3241595	CHEMBL203	IC50	CHEMBL3237931	6.28
CHEMBL3241595	CHEMBL203	IC50	CHEMBL3237932	5.00
CHEMBL3241595	CHEMBL203	IC50	CHEMBL3237934	5.13
CHEMBL3241595	CHEMBL203	IC50	CHEMBL3237935	6.16
CHEMBL3241595	CHEMBL203	IC50	CHEMBL3237936	5.62
CHEMBL3241595	CHEMBL203	IC50	CHEMBL3237937	6.70
CHEMBL3241595	CHEMBL203	IC50	CHEMBL939	10.00

Supplementary Table 2: Summary about the characteristics and problems of currently available compound activity data from four aspects.

Aspect	Characteristic or problem	Example(s) or description
Data sources	Multiple data sources	Wet-lab experiments, scientific literature, patents
Data distribution	Different compound distributions	Congeneric compounds, compound library
Data distribution	Biased protein exposure	Well-studied proteins, novel proteins
Data distribution	Sparse label matrix	Measured affinities, unmeasured affinities
Data distribution	Distinct label distributions	More positive samples, more negative samples
Source of bias	Batch effect	Experimental error
Source of bias	Measurement bias	IC_{50}, K_i, K_d, potency
Source of bias	Bulk evaluation bias	Co-founding factors
Application scenarios	Multiple stages in drug discovery	Hit identification, hit-to-lead optimization, drug repurposing

Supplementary Table 3: Statistics of the six tasks in CARA.

Task type	VS			LO		
Target type	All	Kinase	GPCR	All	Kinase	GPCR
# Assays	12,029	2,733	2,256	81,187	11,276	22,917
# Proteins	2,242	434	268	4,456	487	579
# Compounds	317,855	25,943	41,352	625,099	111,279	161,263
# Samples	1,237,256	84,605	70,179	1,187,136	200,800	321,904
# Training assays	9,408	1,459	1,584	81,033	11,220	22,872
# Test assays	96	58	18	100	54	43

Supplementary Table 4: Comparison of different benchmark datasets of compound activities.

Dataset	Characteristics					Source
	No decoys[1]?	Sparse labels[2]?	Large scale[3]?	Assay view[4]?	VS & LO?	
Davis	✓					[2]
KIBA	✓	✓	✓			[3]
DUD-E		✓	✓			[21]
MUV		✓		✓		[22]
BindingDB	✓	✓	✓			[4, 23]
Human		✓	✓			[24]
C.elegans		✓	✓			[24]
PDBbind	✓	✓				[1, 25]
FS-Mol	✓	✓	✓	✓		[26]
CARA	✓	✓	✓	✓	✓	This work

[1] Decoys are negative samples generated without experimental validation.
[2] Whether the compound-target matrix is sparse.
[3] A dataset is large-scale if the number of compounds, the number of proteins, and the number of compounds per protein are all greater than 100.
[4] Whether the activity samples are organized into assays or not.

Supplementary Table 5: Compound activity prediction methods evaluated on the CARA benchmark. MLP: multi-layer perceptron, CNN: convolutional neural network, GNN: graph neural network, RF: random forest, SVM: support vector machine, GBT: gradient boosting tree.

Methods	Compound		Protein		Features from pre-training	Reference
	Input	Architecture	Input	Architecture		
DeepCPI	Fingerprint	MLP	Sequence	MLP	Yes	[9]
DeepConvDTI	Fingerprint	MLP	Sequence	CNN	No	[11]
DeepDTA	SMILES	CNN	Sequence	CNN	No	[10]
Tsubaki	Graph	GNN	Sequence	CNN	No	[14]
GraphDTA	Graph	GNN	Sequence	CNN	No	[12]
MONN	Graph	GNN	Sequence	CNN	No	[13]
TransformerCPI	SMILES	Attention	Sequence	Attention	Yes	[16]
MolTrans	SMILES	Attention	Sequence	Attention	Yes	[15]
MTDNN	Fingerprint	MLP	None	None	No	[27]
RF	Fingerprint	RF	None	None	No	[17]
SVM	Fingerprint	SVM	None	None	No	[19]
GBT	Fingerprint	GBT	None	None	No	[18]
DNN	Fingerprint	MLP	None	None	No	[20]

Supplementary Table 6: Detailed performances of different compound activity prediction models under the zero-shot scenario. For the VS tasks, the per-assay metrics including the enrichment factors at top 1% (EFs@1%) or 5% (EFs@5%) and success rates at top 1% (SRs@1%) or 5% (SRs@5%) were computed. For the LO tasks, the per-assay metrics including Spearman's correlation coefficients (SCCs), Pearman's correlation coefficients (PCCs), and success rates with PCC $>$ 0.5 (SRs@0.5) were computed. Detailed definitions of these metrics can be found in the main text. The standard deviations over five repeats are shown. The best score for each task and each metric is shown in bold and underlined, and those scores that had no significant difference from the best one are also shown in bold.

Method	VS-All task				LO-All task		
	EF@1%	SR@1%	EF@5%	SR@5%	SCC	PCC	SR@0.5
DeepConvDTI	**9.64 ± 1.19**	**39.17 ± 3.06**	**3.30 ± 0.24**	**81.25 ± 2.64**	**0.30 ± 0.01**	**0.31 ± 0.01**	**26.60 ± 2.15**
DeepDTA	**9.09 ± 1.65**	**36.25 ± 4.03**	**3.45 ± 0.45**	**82.71 ± 2.99**	**0.28 ± 0.01**	**0.30 ± 0.01**	22.40 ± 1.36
DeepCPI	**8.03 ± 0.36**	31.88 ± 2.34	3.03 ± 0.22	**78.75 ± 2.76**	0.24 ± 0.01	0.25 ± 0.01	16.00 ± 0.63
MONN	7.27 ± 0.73	32.71 ± 2.84	2.76 ± 0.49	75.62 ± 4.20	0.25 ± 0.01	0.27 ± 0.01	15.40 ± 2.24
Tsubaki	6.18 ± 1.54	30.00 ± 3.86	2.58 ± 0.15	**78.75 ± 2.99**	0.19 ± 0.02	0.19 ± 0.01	9.40 ± 1.62
TransformerCPI	5.72 ± 0.51	27.71 ± 2.76	2.51 ± 0.29	**77.50 ± 2.34**	0.19 ± 0.01	0.19 ± 0.02	8.00 ± 2.90
MolTrans	5.80 ± 0.89	29.38 ± 2.22	2.23 ± 0.14	72.92 ± 1.86	0.20 ± 0.01	0.20 ± 0.02	12.20 ± 1.47
GraphDTA	4.85 ± 0.97	24.17 ± 2.41	1.92 ± 0.22	70.42 ± 4.20	0.22 ± 0.01	0.24 ± 0.01	15.20 ± 2.04
Method	VS-Kinase task				LO-Kinase task		
	EF@1%	SR@1%	EF@5%	SR@5%	SCC	PCC	SR@0.5
DeepConvDTI	**17.30 ± 2.59**	**42.07 ± 4.70**	**5.37 ± 0.33**	**91.03 ± 3.99**	**0.37 ± 0.01**	**0.38 ± 0.01**	**37.78 ± 1.89**
DeepDTA	**14.44 ± 1.12**	**38.97 ± 2.80**	4.39 ± 0.51	**90.00 ± 4.42**	0.32 ± 0.01	0.33 ± 0.01	27.78 ± 3.10
DeepCPI	12.35 ± 2.36	32.41 ± 5.60	4.02 ± 0.26	**86.21 ± 5.67**	0.31 ± 0.01	0.32 ± 0.01	25.19 ± 2.22
MONN	**14.61 ± 1.22**	**40.34 ± 2.07**	4.62 ± 0.18	**91.72 ± 2.97**	0.28 ± 0.01	0.28 ± 0.01	22.22 ± 2.62
Tsubaki	**13.52 ± 0.75**	**37.59 ± 2.29**	4.14 ± 0.27	87.93 ± 1.89	0.24 ± 0.02	0.25 ± 0.02	17.41 ± 3.23
TransformerCPI	**12.42 ± 1.19**	33.79 ± 3.38	3.86 ± 0.47	**84.83 ± 3.34**	0.22 ± 0.02	0.23 ± 0.01	12.22 ± 2.51
MolTrans	**13.66 ± 1.80**	**37.59 ± 3.99**	4.34 ± 0.20	**90.34 ± 2.80**	0.22 ± 0.01	0.23 ± 0.01	14.44 ± 1.81
GraphDTA	**13.40 ± 1.36**	**36.21 ± 3.27**	3.75 ± 0.15	84.14 ± 2.01	0.24 ± 0.02	0.25 ± 0.02	21.11 ± 0.91
Method	VS-GPCR task				LO-GPCR task		
	EF@1%	SR@1%	EF@5%	SR@5%	SCC	PCC	SR@0.5
DeepConvDTI	**7.91 ± 4.33**	**30.00 ± 9.69**	**2.92 ± 0.66**	70.00 ± 6.67	**0.28 ± 0.01**	**0.29 ± 0.01**	**25.58 ± 4.16**
DeepDTA	**3.79 ± 1.93**	**23.33 ± 4.16**	**2.82 ± 0.69**	66.67 ± 7.03	**0.26 ± 0.02**	**0.26 ± 0.02**	18.14 ± 1.74
DeepCPI	**3.28 ± 2.29**	**18.89 ± 5.67**	**2.22 ± 0.20**	61.11 ± 0.00	0.24 ± 0.01	0.25 ± 0.02	14.88 ± 2.37
MONN	**4.60 ± 4.82**	**22.22 ± 9.94**	**2.57 ± 0.71**	**71.11 ± 5.44**	**0.24 ± 0.01**	0.25 ± 0.01	7.91 ± 4.31
Tsubaki	**6.43 ± 2.10**	**27.78 ± 3.51**	**2.35 ± 0.26**	57.78 ± 4.44	0.16 ± 0.02	0.17 ± 0.02	4.65 ± 3.89
TransformerCPI	**7.68 ± 1.55**	**28.89 ± 5.44**	**3.12 ± 0.23**	**78.89 ± 4.16**	0.18 ± 0.03	0.19 ± 0.03	4.19 ± 1.74
MolTrans	**6.48 ± 4.14**	**23.33 ± 6.48**	**2.50 ± 0.46**	**71.11 ± 5.44**	0.17 ± 0.01	0.17 ± 0.01	6.05 ± 3.78
GraphDTA	**4.01 ± 1.96**	**23.33 ± 2.22**	**2.38 ± 0.35**	67.78 ± 4.16	0.20 ± 0.03	0.22 ± 0.03	8.84 ± 1.74

Supplementary Table 7: Detailed performances of different compound activity prediction models trained on the VS-All (LO-All) data and evaluated on the VS-Kinase (LO-Kinase) or VS-GPCR (LO-GPCR) data under the zero-shot scenario. For the VS tasks (i.e., on the VS-Kinase and VS-GPCR test data), the per-assay metrics including enrichment factors at top 1% (EFs@1%) or 5% (EFs@5%) and success rates at top 1% (SRs@1%) or 5% (SRs@5%) were computed. For the LO tasks (i.e., on the LO-Kinase and LO-GPCR test data), the per-assay metrics including Spearman's correlation coefficients (SCCs), Pearman's correlation coefficients (PCCs), and success rates with PCC $>$ 0.5 (SRs@0.5) were computed. The definitions of these metrics can be found in the main text. The standard deviations over five repeats are shown.

Method	VS-Kinase task				LO-Kinase task		
	EF@1%	SR@1%	EF@5%	SR@5%	SCC	PCC	SR@0.5
DeepConvDTI	17.30 ± 2.59	42.07 ± 4.70	5.37 ± 0.33	91.03 ± 3.99	0.37 ± 0.01	0.38 ± 0.01	37.78 ± 1.89
DeepDTA	14.44 ± 1.12	38.97 ± 2.80	4.39 ± 0.51	90.00 ± 4.42	0.32 ± 0.01	0.33 ± 0.01	27.78 ± 3.10
DeepCPI	12.35 ± 2.36	32.41 ± 5.60	4.02 ± 0.26	86.21 ± 5.67	0.31 ± 0.01	0.32 ± 0.01	25.19 ± 2.22
MONN	14.61 ± 1.22	40.34 ± 2.07	4.62 ± 0.18	91.72 ± 2.97	0.28 ± 0.01	0.28 ± 0.01	22.22 ± 2.62
Tsubaki	13.52 ± 0.75	37.59 ± 2.29	4.14 ± 0.27	87.93 ± 1.89	0.24 ± 0.02	0.25 ± 0.02	17.41 ± 3.23
TransformerCPI	12.42 ± 1.19	33.79 ± 3.38	3.86 ± 0.47	84.83 ± 3.34	0.22 ± 0.02	0.23 ± 0.01	12.22 ± 2.51
MolTrans	13.66 ± 1.80	37.59 ± 3.99	4.34 ± 0.20	90.34 ± 2.80	0.22 ± 0.01	0.23 ± 0.01	14.44 ± 1.81
GraphDTA	13.40 ± 1.36	36.21 ± 3.27	3.75 ± 0.15	84.14 ± 2.01	0.24 ± 0.02	0.25 ± 0.02	21.11 ± 0.91
Method	VS-GPCR task				LO-GPCR task		
	EF@1%	SR@1%	EF@5%	SR@5%	SCC	PCC	SR@0.5
DeepConvDTI	7.91 ± 4.33	30.00 ± 9.69	2.92 ± 0.66	70.00 ± 6.67	0.28 ± 0.01	0.29 ± 0.01	25.58 ± 4.16
DeepDTA	3.79 ± 1.93	23.33 ± 4.16	2.82 ± 0.69	66.67 ± 7.03	0.26 ± 0.02	0.26 ± 0.02	18.14 ± 1.74
DeepCPI	3.28 ± 2.29	18.89 ± 5.67	2.22 ± 0.20	61.11 ± 0.00	0.24 ± 0.01	0.25 ± 0.02	14.88 ± 2.37
MONN	4.60 ± 4.82	22.22 ± 9.94	2.57 ± 0.71	71.11 ± 5.44	0.24 ± 0.01	0.25 ± 0.01	7.91 ± 4.31
Tsubaki	6.43 ± 2.10	27.78 ± 3.51	2.35 ± 0.26	57.78 ± 4.44	0.16 ± 0.02	0.17 ± 0.02	4.65 ± 3.89
TransformerCPI	7.68 ± 1.55	28.89 ± 5.44	3.12 ± 0.23	78.89 ± 4.16	0.18 ± 0.03	0.19 ± 0.03	4.19 ± 1.74
MolTrans	6.48 ± 4.14	23.33 ± 6.48	2.50 ± 0.46	71.11 ± 5.44	0.17 ± 0.01	0.17 ± 0.01	6.05 ± 3.78
GraphDTA	4.01 ± 1.96	23.33 ± 2.22	2.38 ± 0.35	67.78 ± 4.16	0.20 ± 0.03	0.22 ± 0.03	8.84 ± 1.74

Supplementary Table 8: Detailed performances of different compound activity prediction strategies under the few-shot scenario. For the VS tasks, the per-assay metrics including enrichment factors at top 1% (EFs@1%) and success rates at top 1% (SRs@1%) were computed. For the LO tasks, the per-assay metrics including Pearman's correlation coefficients (PCCs), and success rates with PCC $>$ 0.5 (SRs@0.5) were computed. Detailed definitions of these metrics can be found in the main text. The standard deviations over five repeats are shown. The best score for each task and each metric is shown in bold and underlined, and those scores that had no significant difference from the best one are also shown in bold.

Strategy	Method	VS-All task		LO-All task	
		EF@1%	SR@1%	PCC	SR@0.5
Pre-training	DeepCPI	8.26 ± 0.85	29.79 ± 1.69	0.26 ± 0.01	16.60 ± 1.02
Pre-training	DeepDTA	9.51 ± 1.64	**34.38 ± 4.80**	0.30 ± 0.01	24.00 ± 1.67
Pre-training	DeepConvDTI	9.28 ± 1.31	35.00 ± 3.13	0.32 ± 0.01	28.60 ± 1.85
QSAR	RF	7.98 ± 0.55	27.50 ± 1.06	0.55 ± 0.00	58.20 ± 1.72
QSAR	GBT	8.01 ± 0.59	27.92 ± 1.02	0.54 ± 0.00	57.80 ± 1.17
QSAR	SVM	6.40 ± 0.00	27.08 ± 0.00	**0.57 ± 0.00**	**65.00 ± 0.00**
QSAR	DNN	9.13 ± 0.39	26.88 ± 1.02	0.54 ± 0.00	61.20 ± 1.72
Pre-training and fine-tuning	DeepCPI	10.32 ± 0.89	33.12 ± 1.21	0.33 ± 0.01	18.80 ± 0.40
Pre-training and fine-tuning	DeepDTA	**13.45 ± 3.39**	**41.46 ± 5.53**	0.49 ± 0.01	51.00 ± 1.79
Pre-training and fine-tuning	DeepConvDTI	11.45 ± 2.07	36.67 ± 3.12	0.39 ± 0.01	32.40 ± 1.36
Meta-learning	DeepCPI-c	12.19 ± 2.17	**36.46 ± 2.95**	0.42 ± 0.02	37.40 ± 3.44
Meta-learning	MTDNN	**12.89 ± 0.38**	**40.42 ± 1.21**	0.46 ± 0.04	42.26 ± 6.92
Meta-learning	DeepConvDTI-c	**16.25 ± 1.69**	**44.38 ± 3.88**	0.55 ± 0.01	**62.80 ± 4.07**
Multi-task learning	MTDNN	4.31 ± 1.73	25.00 ± 5.15	0.48 ± 0.00	48.20 ± 1.60
Multi-task learning	DeepConvDTI-c	**16.36 ± 1.87**	**44.58 ± 2.59**	**0.58 ± 0.00**	**65.40 ± 2.06**
Re-training	DeepCPI	**13.60 ± 1.46**	**40.62 ± 2.80**	0.44 ± 0.00	43.89 ± 2.91
Re-training	DeepDTA	12.26 ± 2.16	**39.38 ± 2.90**	0.46 ± 0.01	45.80 ± 1.72
Re-training	DeepConvDTI	**13.81 ± 0.63**	**43.33 ± 2.14**	0.50 ± 0.01	54.40 ± 1.96

Supplementary Table 9: Statistics of the selected assays for in-distribution (ID) or out-of-distribution (OOD) training. A sample stands for a pair of a compound and a protein with a measured activity label.

Task type	VS			LO		
Subset	All	ID	OOD	All	ID	OOD
# Training assays	9,408	4,526	5,900	81,033	5,482	2,109
# Test assays	96	39	39	100	37	37
# Training samples	777,987	155,598	155,598	1,137,015	35,103	35,103
# Test samples	324,085	14,776	14,776	24,588	7,192	7,192

Supplementary Table 10: Statistics of the pharmacological profiles of test assays in our CARA benchmark.

Task type	Binding	Agonism/activation	Antagonism/inhibition	Others	Total
VS-All	5	6	84	1	96
VS-Kinase	0	0	58	0	58
VS-GPCR	8	4	6	0	18
LO-All	14	7	79	0	100
LO-Kinase	0	0	54	0	54
LO-GPCR	24	9	10	0	43

Supplementary Table 11: Statistics of the unexplored test compounds in test assays of our CARA benchmark. The test assays containing at least 50% of unexplored compounds are shown in the table.

Task type	Assay ID	Unexplored (%)[1]	Unexplored (#)[2]	Total (#)[3]
VS-All	CHEMBL1827362_IC50	78.74 %	163	207
VS-All	CHEMBL2038597_IC50	69.88 %	116	166
VS-All	CHEMBL2038446_IC50	69.88 %	116	166
VS-All	CHEMBL1826790_IC50	64.96 %	291	448
VS-All	CHEMBL4649962_IC50	64.23 %	246	383
LO-All	CHEMBL3887456_EC50	100.00 %	230	230
LO-All	CHEMBL3888209_IC50	99.72 %	361	362
LO-All	CHEMBL3888033_IC50	99.59 %	242	243
LO-All	CHEMBL3705332_IC50	99.34 %	151	152
LO-All	CHEMBL819742_Ki	98.09 %	154	157
LO-All	CHEMBL3887259_EC50	91.34 %	232	254
LO-All	CHEMBL3705963_IC50	90.30 %	149	165
LO-All	CHEMBL4418470_EC50	88.30 %	249	282
LO-All	CHEMBL4739819_EC50	77.09 %	461	598
LO-All	CHEMBL3705480_Ki	77.03 %	285	370
LO-All	CHEMBL4476912_IC50	72.65 %	85	117
LO-All	CHEMBL3705476_IC50	68.58 %	454	662
LO-All	CHEMBL3888296_IC50	67.83 %	97	143
LO-All	CHEMBL3705983_IC50	66.50 %	268	403
LO-All	CHEMBL3707783_IC50	63.35 %	102	161
LO-All	CHEMBL3887078_IC50	54.62 %	71	130
LO-All	CHEMBL3706356_IC50	53.77 %	114	212

[1] Percentage of unexplored test compounds.
[2] Number of unexplored test compounds.
[3] Total number of test compounds.

Supplementary References

[1] Renxiao Wang, Xueliang Fang, Yipin Lu, Chao-Yie Yang, and Shaomeng Wang. The PDBbind database: methodologies and updates. *Journal of Medicinal Chemistry*, 48(12):4111–4119, 2005.

[2] Mindy I Davis, Jeremy P Hunt, Sanna Herrgard, Pietro Ciceri, Lisa M Wodicka, Gabriel Pallares, Michael Hocker, Daniel K Treiber, and Patrick P Zarrinkar. Comprehensive analysis of kinase inhibitor selectivity. *Nature Biotechnology*, 29(11):1046–1051, 2011.

[3] Jing Tang, Agnieszka Szwajda, Sushil Shakyawar, Tao Xu, Petteri Hintsanen, Krister Wennerberg, and Tero Aittokallio. Making sense of large-scale kinase inhibitor bioactivity data sets: a comparative and integrative analysis. *Journal of Chemical Information and Modeling*, 54(3):735–743, 2014.

[4] Tiqing Liu, Yuhmei Lin, Xin Wen, Robert N Jorissen, and Michael K Gilson. BindingDB: a web-accessible database of experimentally determined protein–ligand binding affinities. *Nucleic Acids Research*, 35(suppl_1):D198–D201, 2007.

[5] David S Wishart, Yannick D Feunang, An C Guo, Elvis J Lo, Ana Marcu, Jason R Grant, Tanvir Sajed, Daniel Johnson, Carin Li, Zinat Sayeeda, et al. Drugbank 5.0: a major update to the drugbank database for 2018. *Nucleic Acids Research*, 46(D1):D1074–D1082, 2018.

[6] Enamine Ltd. Enamine HTS collection. https://enamine.net/compound-collections/screening-collection/hts-collection, 2023.

[7] John J Irwin and Brian K Shoichet. ZINC- a free database of commercially available compounds for virtual screening. *Journal of Chemical Information and Modeling*, 45(1):177–182, 2005.

[8] Xian Zeng, Peng Zhang, Weidong He, Chu Qin, Shangying Chen, Lin Tao, Yali Wang, Ying Tan, Dan Gao, Bohua Wang, et al. NPASS: natural product activity and species source database for natural product research, discovery and tool development. *Nucleic Acids Research*, 46(D1):D1217–D1222, 2018.

[9] Fangping Wan, Yue Zhu, Hailin Hu, Antao Dai, Xiaoqing Cai, Ligong Chen, Haipeng Gong, Tian Xia, Dehua Yang, Ming-Wei Wang, et al. DeepCPI: A deep learning-based framework for large-scale in silico drug screening. *Genomics, Proteomics & Bioinformatics*, 17(5):478–495, 2019.

[10] Hakime Öztürk, Arzucan Özgür, and Elif Ozkirimli. DeepDTA: deep drug–target binding affinity prediction. *Bioinformatics*, 34(17):i821–i829, 2018.

[11] Ingoo Lee, Jongsoo Keum, and Hojung Nam. DeepConv-DTI: Prediction of drug-target interactions via deep learning with convolution on protein sequences. *PLoS Computational Biology*, 15(6):e1007129, 2019.

[12] Thin Nguyen, Hang Le, Thomas P Quinn, Tri Nguyen, Thuc Duy Le, and Svetha Venkatesh. GraphDTA: Predicting drug–target binding affinity with graph neural networks. *Bioinformatics*, 37(8):1140–1147, 2021.

[13] Shuya Li, Fangping Wan, Hantao Shu, Tao Jiang, Dan Zhao, and Jianyang Zeng. MONN: a multi-objective neural network for predicting compound-protein interactions and affinities. *Cell Systems*, 10(4):308–322, 2020.

[14] Masashi Tsubaki, Kentaro Tomii, and Jun Sese. Compound–protein interaction prediction with end-to-end learning of neural networks for graphs and sequences. *Bioinformatics*, 35(2):309–318, 2019.

[15] Kexin Huang, Cao Xiao, Lucas M Glass, and Jimeng Sun. MolTrans: Molecular interaction transformer for drug–target interaction prediction. *Bioinformatics*, 37(6):830–836, 2021.

[16] Lifan Chen, Xiaoqin Tan, Dingyan Wang, Feisheng Zhong, Xiaohong Liu, Tianbiao Yang, Xiaomin Luo, Kaixian Chen, Hualiang Jiang, and Mingyue Zheng. TransformerCPI: improving compound–protein interaction prediction by sequence-based deep learning with self-attention mechanism and label reversal experiments. *Bioinformatics*, 36(16):4406–4414, 2020.

[17] Tin Kam Ho. Random decision forests. In *Proceedings of 3rd International Conference on Document Analysis and Recognition*, volume 1, pages 278–282. IEEE, 1995.

[18] Jerome H Friedman. Greedy function approximation: a gradient boosting machine. *Annals of Statistics*, pages 1189–1232, 2001.

[19] Corinna Cortes and Vladimir Vapnik. Support-vector networks. *Machine learning*, 20(3):273–297, 1995.

[20] Simon Haykin. *Neural Networks: a Comprehensive Foundation*. Prentice Hall PTR, 1994.

[21] Michael M Mysinger, Michael Carchia, John J Irwin, and Brian K Shoichet. Directory of useful decoys, enhanced (DUD-E): better ligands and decoys for better benchmarking. *Journal of Medicinal Chemistry*, 55(14):6582–6594, 2012.

[22] Sebastian G Rohrer and Knut Baumann. Maximum unbiased validation (MUV) data sets for virtual screening based on pubchem bioactivity data. *Journal of Chemical Information and Modeling*, 49(2):169–184, 2009.

[23] Michael K Gilson, Tiqing Liu, Michael Baitaluk, George Nicola, Linda Hwang, and Jenny Chong. BindingDB in 2015: a public database for medicinal chemistry, computational chemistry and systems pharmacology. *Nucleic Acids Research*, 44(D1):D1045–D1053, 2016.

[24] Hui Liu, Jianjiang Sun, Jihong Guan, Jie Zheng, and Shuigeng Zhou. Improving compound–protein interaction prediction by building up highly credible negative samples. *Bioinformatics*, 31(12):i221–i229, 2015.

[25] Zhihai Liu, Yan Li, Li Han, Jie Li, Jie Liu, Zhixiong Zhao, Wei Nie, Yuchen Liu, and Renxiao Wang. PDB-wide collection of binding data: current status of the PDBbind database. *Bioinformatics*, 31(3):405–412, 2015.

[26] Megan Stanley, John F Bronskill, Krzysztof Maziarz, Hubert Misztela, Jessica Lanini, Marwin Segler, Nadine Schneider, and Marc Brockschmidt. Fs-mol: A few-shot learning dataset of molecules. In *Thirty-fifth Conference on Neural Information Processing Systems Datasets and Benchmarks Track (Round 2)*, 2021.

[27] Xutong Li, Zhaojun Li, Xiaolong Wu, Zhaoping Xiong, Tianbiao Yang, Zunyun Fu, Xiaohong Liu, Xiaoqin Tan, Feisheng Zhong, Xiaozhe Wan, et al. Deep learning enhancing kinome-wide polypharmacology profiling: model construction and experiment validation. *Journal of Medicinal Chemistry*, 63(16):8723–8737, 2019.
