# Supplementary material for: Benchmarking compound activity prediction for real-world drug discovery applications
Source: Commun Chem. 2024 Jun 4;7:127. doi: 10.1038/s42004-024-01204-4 (PMC11150475; doi:10.1038/s42004-024-01204-4)
Supplement: Supplementary file 2 — Description of Additional Supplementary Files [file 42004_2024_1204_MOESM2_ESM.pdf]

Description of Additional Supplementary Files:

File name- Supplementary Data 1

File description- Pharmacological profiles measured by different assays.

File name- Supplementary Data 2

File description- Source data of main figures.
